# Kynurenine 3-Monooxygenase: An Influential Mediator of Neuropathology

**DOI:** 10.3389/fpsyt.2015.00116

**Published:** 2015-08-20

**Authors:** Jennifer M. Parrott, Jason C. O’Connor

**Affiliations:** ^1^Department of Pharmacology, School of Medicine, University of Texas Health Science Center at San Antonio, San Antonio, TX, USA; ^2^Center for Biomedical Neuroscience, University of Texas Health Science Center at San Antonio, San Antonio, TX, USA; ^3^Mood Disorders Translational Research Core, University of Texas Health Science Center at San Antonio, San Antonio, TX, USA; ^4^Audie L. Murphy Memorial VA Hospital, South Texas Veterans Health System, San Antonio, TX, USA

**Keywords:** neuroinflammation, kynurenine 3-monooxygenase, kynurenine pathway, microglia, neurodevelopmental disorders, neurodegenerative diseases, neuropsychiatric disorders

## Abstract

Mounting evidence demonstrates that kynurenine metabolism may play an important pathogenic role in the development of multiple neurological and neuropsychiatric disorders. The kynurenine pathway consists of two functionally distinct branches that generate both neuroactive and oxidatively reactive metabolites. In the brain, the rate-limiting enzyme for one of these branches, kynurenine 3-monooxygenase (KMO), is predominantly expressed in microglia and has emerged as a pivotal point of metabolic regulation. KMO substrate and expression levels are upregulated by pro-inflammatory cytokines and altered by functional genetic mutations. Increased KMO metabolism results in the formation of metabolites that activate glutamate receptors and elevate oxidative stress, while recent evidence has revealed neurodevelopmental consequences of reduced KMO activity. Together, the evidence suggests that KMO is positioned at a critical metabolic junction to influence the development or trajectory of a myriad of neurological diseases. Understanding the mechanism(s) by which alterations in KMO activity are able to impair neuronal function, and viability will enhance our knowledge of related disease pathology and provide insight into novel therapeutic opportunities. This review will discuss the influence of KMO on brain kynurenine metabolism and the current understanding of molecular mechanisms by which altered KMO activity may contribute to neurodevelopment, neurodegenerative, and neuropsychiatric diseases.

## Introduction

**Figure 1:**
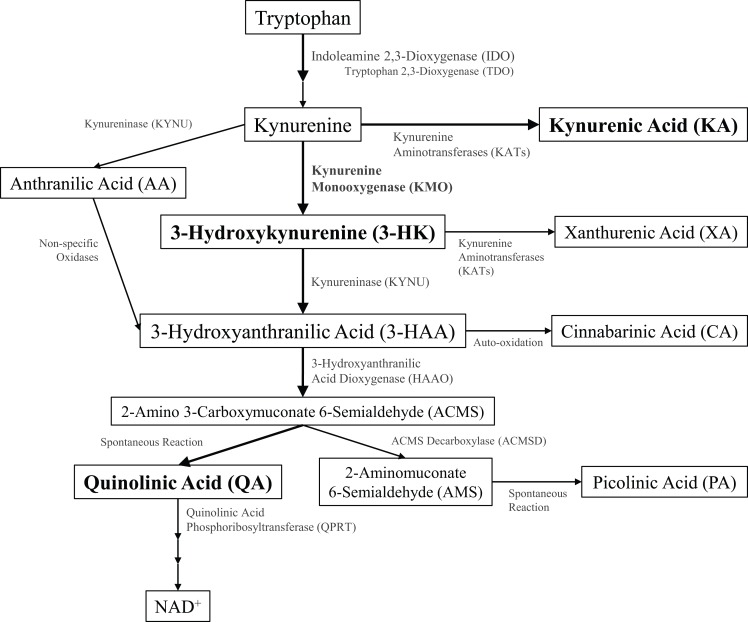
**Schematic of kynurenine pathway metabolism**. Kynurenine metabolites (inside boxes) and the enzymes that metabolize them (along arrows) are illustrated.

During healthy conditions, most dietary tryptophan is metabolized in the liver to generate nicotinic acid and subsequent energy-producing co-factors through the kynurenine metabolic pathway (1). Kynurenine metabolites were first identified in urinary excretions, and the observation of a correlation between the disruption of dietary tryptophan metabolism and neuropsychiatric diseases sparked an interest in the potential pathogenic role of kynurenines (2, 3). Identification of receptor targets within the brain provided a putative mechanistic role for kynurenine metabolites in disorders of the central nervous system. The kynurenine pathway (Figure 1) diverges along two main metabolic branches that produce metabolites, kynurenic acid (KA), and quinolinic acid (QA), that both bind to the *N*-methyl-d-aspartate receptor (NMDAR) (4). KA or QA modulation of NMDAR activity and glutamatergic signaling is hypothesized to contribute to the pathogenesis of multiple neurological disorders (5). Under basal conditions, kynurenine metabolism favors the formation of KA within the brain; however, disruptions in homeostasis can shift the balance toward increased production of QA. The rate-limiting step for the production of QA involves oxidation of kynurenine by kynurenine 3-monooxygenase (KMO). As KMO is positioned at a pivotal junction in regulating the production of these two metabolites, changes in KMO expression or activity may contribute to the development of neurodegenerative, neuropsychiatric, and neurodevelopmental diseases. The purpose of this review is to (1) provide evidence of the critical role that KMO plays in maintaining the physiological balance between QA and KA production, (2) present examples of how this balance can be disrupted and associated with neurological diseases, and (3) demonstrate how this evidence supports KMO as a potential therapeutic target for neurological disorders.

## Kynurenine Metabolism in the Brain

Tryptophan is metabolized to kynurenine by one of three rate-limiting enzymes; tryptophan 2,3-dioxygenase (TDO), indoleamine-2,3-dioxygenase (IDO1), or indoleamine-2,3-dioxygenase-like protein (IDO2). TDO expression increases in response to substrate levels or corticosteroid stress hormones, while inflammatory stimuli increase IDO1 expression (6, 7). Fluctuations in kynurenine metabolism have the capacity to indicate changes in homeostasis in the body. Under normal physiological conditions, local production of kynurenine within the brain accounts for approximately one-third of the kynurenine present, while the rest is transported in from the periphery (8). During disease conditions, however, an even larger proportion of brain kynurenine originates from peripheral sources, and the subsequent synthesis of neuroactive kynurenine metabolites also increase and have the capacity to influence neurotransmitter systems within the brain (9).

### Kynurenine

Until recently, kynurenine, itself, was considered a biologically inert metabolite. Kynurenine levels and the kynurenine to tryptophan ratio were largely evaluated for their utility as biomarkers for disease. Early hypotheses suggested that increased tryptophan metabolism along the kynurenine pathway could reduce the bioavailability of tryptophan for the synthesis of serotonin resulting in depression-associated behaviors (10). Recent clinical and preclinical data, within the context of inflammation, indicate that the brain is able to compensate for reduced peripheral tryptophan levels leaving central serotonin levels unaffected (11, 12). However, identification of kynurenine as an endothelium-derived vasodilator revealed a more relevant physiological role for this metabolite (13). The subsequent discovery of kynurenine as an endogenous ligand of the aryl hydrocarbon receptor has prompted intense interest into this potential receptor-mediated mechanism by which kynurenine could contribute directly to disease (14). Further, kynurenine is readily transported across the blood–brain barrier, and is the substrate for the synthesis of many metabolites that exert known neurotoxic, neuroactive, and oxidative actions, which have been implicated in a wide range of neuropathologies.

### Kynurenic acid

Under physiological conditions, most kynurenine in the brain is metabolized to KA (Figure 1), an NMDAR antagonist, and α_7_-nicotinic acetylcholine receptor (α_7_nAChR)-negative allosteric modulator (4, 15). Peripheral KA does not cross the blood–brain barrier in appreciable amounts and therefore is not thought to contribute to central actions of KA. Kynurenine aminotransferase (KAT) catalyzes this reaction and is not responsive to inflammatory stimuli as other kynurenine pathway enzymes are. There are three functional KAT isoforms, I and II the most relevant in mammals, and are predominantly expressed in astrocytes, giving rise to cell-type specific compartmentalization kynurenine metabolism (16, 17). Early studies of KA demonstrated that it can be neuroprotective against neuronal damage caused by neurotoxic QA, an effect that is most likely mediated through inhibitory activity at NMDARs (18). More recent studies have demonstrated that elevated KA can lead to disruptions in working memory, sensorimotor gating, and attentional processing tasks (19–21). These results suggest that sustaining physiological levels of KA are likely important in maintaining a basal neuroprotective environment within the brain, but pathophysiological elevation of KA can also be detrimental to normal neuronal functioning.

### 3-hydroxykynurenine

During inflammatory conditions and when IDO1 is upregulated, kynurenine metabolism shifts from the predominant production of KA toward the generation of increased amounts of QA. In the first metabolic reaction of this branch, KMO metabolizes kynurenine to 3-hydroxykynurenine (3-HK, Figure 1). While KAT enzymes are expressed in astrocytes, KMO is predominantly expressed in microglia, the resident immune cells in the brain (22). In response to inflammatory stimuli or tissue damage, KMO expression and 3-HK production also increase, effectively shuttling kynurenine metabolism toward the production of QA (23). Peripherally generated 3-HK is able to cross the blood–brain barrier by virtue of it hydrophobic nature, increasing its bioavailability in the brain. 3-HK was originally thought to be a cytotoxic metabolite through the generation of reactive oxygen species (24–26). However, more recent studies have demonstrated that 3-HK has the ability to be both antioxidant as well as pro-oxidant depending on the circumstances (27). In the brain, 3-HK may function primarily as a modulator of redox state as opposed to being a neurotoxic metabolite, but the direct neuropathological role of 3-HK remains under investigation.

### 3-hydroxyanthranilic acid

Kynureninase (KYNU) metabolizes 3-HK to 3-hydroxyanthranilic acid (3-HAA, Figure 1), another metabolite that is increased during inflammatory conditions (23). 3-HAA has been shown to play a role in T-cell immunity by reducing dendritic cell activation of T-cells and by mediating activated T-cell death (28, 29). Additionally, 3-HAA can induce apoptosis in monocyte-derived cells; however, 3-HAA is also anti-inflammatory and neuroprotective in neuronal and glial cultures (30–32).

### Quinolinic acid

3-Hydroxyanthranilic acid dioxygenase (HAAO) metabolizes 3-HAA into 2-amino 3-carboxymuconate 6-semialdehyde (ACMS, Figure 1), which undergoes a non-enzymatic conversion to QA (Figure 1), an endogenous NMDAR agonist (33). During inflammatory conditions, QA accumulates and is a major endpoint of kynurenine metabolism. With the capacity to stimulate excitatory glutamatergic signaling, extra-physiological concentrations of QA can result in neurotoxicity or neuronal death, tissue lesions, and seizures (33–35). Additionally, QA neurotoxicity is also attributed to the generation of reactive oxygen species and lipid peroxidation (36–38). Due to its potent neurotoxic properties, any elevation or accumulation of QA can have detrimental effects on not only the local cellular environment but also can impact behavior as well.

### Other kynurenine metabolites

#### Anthranilic Acid

Kynurenine can also be metabolized to anthranilic acid (AA, Figure 1) by KYNU. However, the affinity of kynurenine for this enzyme is very low, so metabolism only occurs to appreciable amounts when kynurenine concentrations are elevated (39). AA can also be metabolized by non-specific oxidases to generate 3-HAA, a reaction which does appear to contribute significantly to the production of 3-HAA (40). AA does not increase during inflammatory conditions and no receptor activity for AA has been identified presently (23).

#### Xanthurenic Acid

The transamination of 3-HK results in xanthurenic acid (XA, Figure 1), and this enzymatic reaction is conducted by the same aminotransferase (KAT) that metabolizes kynurenine to KA (41). Within the brain, XA appears to be functionally relevant as it is stored and transported within neuronal vesicles and then released in an activity-dependent manner (41). Further, evidence demonstrates that XA can modulate hippocampal transmission through inhibition of the vesicular glutamate transporter (VGLUT) and was recently identified as an endogenous agonist of metabotropic glutamate receptors (42, 43).

#### Cinnabarinic Acid

Cinnabarinic acid (CA, Figure 1) is produced by the auto-oxidation of two 3-HAA molecules in a process that also generates superoxide and hydrogen peroxide (44). Synthesis of CA occurs during conditions of elevated reactive oxygen species or in response to inflammatory stimuli (45, 46). CA is a partial agonist at type 4 metabotropic glutamate receptors (mGluR4), activation of which can protect neurons from excitotoxic death (46).

#### Picolinic Acid

Rather than be converted to QA, ACMS can be metabolized by ACMS decarboxylase (ACMSD) into 2-aminomuconate 6-semialdehyde (AMS, Figure 1) that can be non-enzymatically converted to picolinic acid (PA, Figure 1) (47). ACMSD expression is low and therefore PA formation does not represent the main route of metabolism of ACMS (47). PA can attenuate QA-induced neurotoxicity through a mechanism independent of influencing neuronal excitation (48).

## Influence of Glia on Central Kynurenine Metabolism

**Figure 2:**
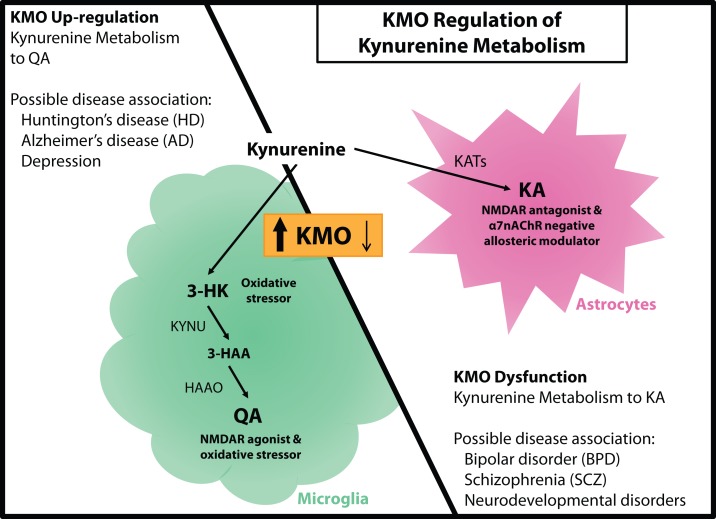
**KMO regulation of kynurenine metabolism**. (Left panel) KMO upregulation during neuroinflammation results in a shift in kynurenine metabolism that increases production of QA in microglia. This change in metabolism is associated with elevated oxidative stress and glutamate excitotoxicity that could contribute to Huntington’s disease (HD), Alzheimer’s disease (AD), and even depression. (Right panel) KMO dysfunction as a consequence of genetic mutation favors the production of KA in astrocytes, which is accompanied by disruptions in cognitive performance and psychosis. KA elevations and KMO polymorphisms have been associated with bipolar disorder (BPD), schizophrenia (SCZ), and some neurodevelopmental disorders. KMO has the potential to regulate the production of both QA and KA thereby contributing to the development of multiple neurological disorders.

Though peripherally produced tryptophan and kynurenine contribute most of the substrate for metabolism in the kynurenine pathway in the brain, KA and QA do not appreciably cross the blood–brain barrier and must be produced locally. Since the enzymes that subsequently direct the metabolism of kynurenine are preferentially expressed in separate cell types, production of these neuroactive metabolites is essentially compartmentalized within the brain and sensitive to inflammatory signals that activate microglia (Figure 2).

While most cells in the brain have the capacity to metabolize tryptophan to kynurenine, metabolism of kynurenine to KA occurs mainly in astrocytes, and the production of QA occurs mainly in activated microglia (22, 49). Under basal conditions in the brain, kynurenine is predominantly metabolized to KA in astrocytes, cells that primarily function to maintain homeostasis within the brain (16). However, during neuroinflammatory conditions, kynurenine metabolism shifts to generating neurotoxic QA and microglia become the primary cellular mediators (23). Neuroinflammation includes the propagation of inflammatory signals and the activation of the resident immune cells in the brain, microglia. Shifting the metabolism of kynurenine from astrocytes toward activated microglia during neuroinflammation corresponds with the alteration in production of KA to QA (i.e., increasing the QA to KA ratio). While astrocytes have the capability to clear extracellular QA and metabolize it to nicotinic acid, they are not able to produce it (22). Further, during neuroinflammatory conditions, the capacity of astrocytes to clear QA may be impaired (50). In disease states, microglia can remain active and neuroinflammation can persist, possibly resulting in production and accumulation of neurotoxic kynurenine metabolites. Compartmentalization provides a mechanism to effectively and physically separate the two distinct branches of kynurenine metabolism.

## KMO is Positioned at an Influential Regulatory Point in the Kynurenine Pathway

Multiple kynurenine metabolites have the capacity to directly or indirectly influence neuronal processes, both in a beneficial or detrimental manner. However, those with potentially the greatest impact are KA and QA as they can influence excitatory glutamatergic signaling by binding the NMDAR. Many neurological disorders are hypothesized to involve disrupted glutamate signaling, and therefore, modulation of KA or QA production could provide a more relevant therapeutic target (5). During neuroinflammatory conditions, KMO is the principle regulator of the metabolic fate of kynurenine to either KA or QA, and as such, it could be a prominent mediator of neuropathology development (Figure 2).

### KMO regulates quinolinic acid production

Kynurenine 3-monooxygenase metabolism of kynurenine increases in response to elevated substrate levels and upregulation in KMO expression, both associated with neuroinflammation, typically characterized by microglia activation, and the propagation of central inflammatory signals. Metabolism continues along the KMO-dependent branch of the pathway producing 3-HK, 3-HAA, and QA, contributing neurotoxic metabolites to this environment of neuroinflammation (23, 51). Though the acute process is typically adaptive, prolonged neuroinflammation or the presence of secondary factors that can exacerbate the neuroinflammatory response (i.e., gene polymorphisms, comorbidity, etc.) often results in maladaptive consequences on normal neuronal functioning (52, 53). Numerous neurological disorders are characterized by chronic inflammation, and their pathology is often associated with microglia activation (54). Consequently, continued neuroimmune stimulation can also result in the production of neurotoxic kynurenine metabolites, 3-HK and QA, that generate reactive oxygen species and increase the potential for glutamate excitotoxicity. *In vivo* administration of 3-HK induces tissue damage that is only attenuated when co-treated with *N*-acetyl-l-cysteine, an antioxidant, not MK-801, an NMDAR antagonist (55). *In vitro* studies have further demonstrated that 3-HK can cause neuronal cell death that is susceptible to glutathione, catalase, and desferrioxamine, antioxidants that demonstrate 3-HK confers its cellular toxicity via the generation of oxidative stress (24–26). However, contradictory studies also demonstrate that 3-HK can reduce markers of oxidative stress, specifically in C6 glioma cells, and protects B-phycoerythrin from peroxyl radical-mediated oxidative damage (56, 57). Despite this evidence, the potential for 3-HK to contribute to oxidative stress and neuronal damage, particularly contributing to disease progression, still remains, as elevated 3-HK levels have been described in neurodegenerative and neuropsychiatric disorders (58).

Quinolinic acid can also induce oxidative damage, specifically lipid peroxidation, which is attenuated by pretreatment with KA or MK-801 (36, 59). Lipid peroxidation produced by QA can also be attenuated by antioxidants, demonstrating that both free radical formation and NMDAR activation contribute to QA-induced oxidative damage (60, 61). As microglia remain activated and continue metabolizing kynurenine, QA production similarly increases and is released into the synapse (62). The breakdown of QA by quinolinic acid phosphoribosyl transferase (QPRT) occurs much slower than the production, therefore QA can rapidly accumulate (63, 64). Elevated QA activates NMDARs, stimulates glutamate release, and prevents glutamate re-uptake by astrocytes (65). As extracellular glutamate concentrations rise from continued release and re-uptake inhibition, the neurons in the synapse can undergo cytotoxic cell death (66). Direct administration of QA is a potent inducer of seizures, a behavioral phenotype of neuronal over-excitation (67). As KA can experimentally attenuate QA excitotoxicity and oxidative damage, the existence of this endogenous antagonism suggests that maintenance of metabolic homeostasis within the brain is important, and, as might be predicted, disruption of metabolic balance could contribute to the underlying pathology of many brain-based diseases. In disorders associated with inflammation and microglia activation, increased KMO-dependent kynurenine metabolism would result in 3-HK and QA accumulation, while a disruption of KMO activity would favor a metabolic shift toward the production of KA. The accumulation in neurotoxic metabolites could in turn contribute oxidative stress and neuronal excitotoxicity. KMO therefore represents a viable therapeutic target for a number of diseases associated with neuroinflammation and neurodegeneration.

### KMO regulates kynurenic acid production

Under physiological circumstances in the brain, a majority of kynurenine is metabolized by KATs to KA, predominantly in astrocytes (49). As KA is an inhibitor of the NMDAR, it was initially thought to be neuroprotective by attenuating over-excitation of glutamatergic neurons (4). Multiple studies demonstrated that as the endogenous antagonist to QA, KA could counteract the damaging and neurotoxic effects of elevations in QA *in vivo* specifically through the NMDAR (4, 18, 68, 69). KA also interacts with the mesocorticolimbic dopamine (DA) area through its antagonism of the NDMAR and therefore has the potential to impact disorders associated with this system (70–72). The inhibitory actions of KA on cholinergic transmission through the α_7_nAChR also indirectly influence the regulation of multiple neurotransmitter systems including both GABA and DA signaling in the striatum (73, 74). Despite the seemingly positive impact KA has on neuronal systems, the beneficial effects of elevated KA only extend so far, as pathophysiological levels have been found to be detrimental. Endogenous central KA can be increased by peripheral administration of kynurenine to rodents and has resulted in disruptions in cognitive performance. Specifically, elevations in KA impair sensorimotor gating, attentional processing of environmental stimuli, spatial working memory, and contextual learning memory (19–21, 75). These behavioral tasks have relevance in neuropsychiatric diseases, such as schizophrenia (SCZ) and bipolar disorder (BPD), characterized by cognitive disruptions and psychosis (76). Though KA can be increased experimentally by administration of kynurenine, recently characterized KMO polymorphisms in the human populations provide a clinical context for elevations in KA contributing to pathophysiology. These genetic alterations in KMO are associated with decreased gene expression and increased KA concentrations in patients suffering from SCZ and BPD (77–80). Together, these data support the notion that deviation of kynurenine metabolic balance too far in either direction may contribute to impaired brain function.

## Neurological Disorders and Disrupted Kynurenine Metabolism

Consequences of a persistent imbalance in kynurenine metabolism can be demonstrated experimentally; however, the relevance of kynurenine metabolites to disease pathogenesis has been suggested in several neurological disease contexts, based on patient post-mortem tissue, plasma, and cerebrospinal fluid (CSF) sampling studies. Determination of kynurenine metabolite concentrations in patients diagnosed with neurological disorders allows for more direct correlations between metabolite measures, pathology development, and symptoms. Moreover, preclinical modeling allows for further exploration into the potential mechanism(s) that might connect kynurenine metabolism and disease pathogenesis. Kynurenine metabolic balance in relevant neurodegenerative, neuropsychiatric, and neurodevelopmental disorders is reviewed below (for summary see Table 1).

**Table 1:** **Neurological diseases with disrupted kynurenine metabolism – a focus on KMO regulated metabolites**.

Disease	Species	Observation	Reference
Huntington’s disease (HD)	Human	↑ KA (post-mortem brain, motor cortex)	(81)
	Human	(n.d.) QA (post-mortem brain, putamen, or frontal cortex)	(82)
	Human	(n.d.) QA (cerebrospinal fluid)	(83)
	Human	↑ 3-HK (post-mortem brain, frontal and temporal cortex)	(84)
	Human	↑ Kynurenine/KA Ratio (post-mortem brain, putamen)	(85)
		↓ KA (cerebrospinal fluid)	
	Human	↓ KA (post-mortem brain, five cortical regions)	(86)
		↓ 3-HK (post-mortem brain, inferior temporal gyrus)	
	Human	↓ KA (cerebrospinal fluid)	(5)
		(n.d.) QA (cerebrospinal fluid)	
	Human	↑ 3-HK (post-mortem brain, frontal and temporal cortex, putamen)	(87)
	Human	↑ KA (post-mortem brain, cerebral cortex)	(88)
		↑ 3-HK (post-mortem brain, cerebral cortex, and striatum)	
	FBV/N mice	↑ KA (brain, cortex, and striatum)	(88)
		↑ 3-HK (brain, cortex, and striatum)	
	Human	↑ 3-HK (Grade 0/1 post-mortem brain, striatum, and frontal cortex)	(89)
		↑ QA (Grade 0/1 post-mortem brain, striatum, and frontal cortex)	
		(n.d.) 3-HK (Grade 2–4 post-mortem brain, striatum, or frontal cortex)	
		(n.d.) QA (Grade 2–4 post-mortem brain, striatum, or frontal cortex)	
		(n.d.) KA (Grade 0–4 post-mortem brain, striatum, or frontal cortex)	
	R6/2 mice	↑ 3-HK with age (brain, cortex, striatum, and cerebellum)	(90)
		(n.d.) QA with age (brain, cortex, striatum and cerebellum)	
		(n.d.) KA (brain, cortex, striatum, and cerebellum)	
	Hdh^Q92^ and Hdh^Q111^ mice	↑ 3-HK (15–17 mo. brain, cortex, striatum, and cerebellum)	(90)
		↑ QA (15–17 mo. brain, cortex, and striatum)	
		(n.d.) KA (brain, cortex, striatum, or cerebellum)	
	YAC128 mice	↑ 3-HK with age (brain, cortex, striatum, and cerebellum)	(90)
		↑ QA with age (brain, cortex, and striatum)	
		(n.d.) KA (brain, cortex, striatum, or cerebellum)	
	R6/2 mice	↑ KMO activity with age (brain, cortex)	(91)
	Htt93Q flies	↑ 3-HK/KA ratio (fly heads)	(92)
	YAC128 mice	↓ 3-HK (3 mo. brain, striatum)	(93)
		↓ QA (3 mo. brain, cerebellum)	
		↑ 3-HK (12 mo. brain, striatum)	
		↑ QA (12 mo. brain, cerebellum)	
Alzheimer’s disease (AD)	Human	(n.d.) QA (post-mortem brain, frontal, temporal, or parietal cortex)	(94)
	Human	(n.d.) QA (post-mortem brain, six cortical regions, hippocampus, or caudate)	(95)
		(n.d.) QA (cerebrospinal fluid)	
	Human	(n.d.) KA (post-mortem brain, four cortical regions, or caudate)	(86)
		(n.d.) 3-HK (post-mortem brain, inferior and middle temporal gyrus, or caudate)	
	Human	↓ KA (cerebrospinal fluid)	(5)
		(n.d.) QA (cerebrospinal fluid)	
	Human	(n.d.) 3-HK (post-mortem brain, temporal cortex)	(87)
	Human	↓ 3-HK (cerebrospinal fluid)	(96)
	Human	↑ KA (post-mortem brain, putamen and caudate nucleus)	(97)
		(n.d.) KA (post-mortem brain, frontal cortex, hippocampus, or cerebellum)	
		(n.d.) 3-HK (post-mortem brain, frontal cortex, hippocampus, cerebellum, putamen, or caudate nucleus)	
	Human	↓ KA (serum and red blood cells)	(98)
	Human	↓ KA (serum)	(99)
		↑ QA (serum)	
	APPtg mice	↑ KA (6 mo. brain, cortex)	(100)
		(n.d.) 3-HK (6 mo. brain, cortex)	
		(n.d.) QA (6 mo. brain, cortex)	
	Human	↑ 3-HK (serum)	(101)
		(n.d.) KA (serum)	
		(n.d.) QA (serum)	
Depression	Human	(n.d.) KA (urinary excretions following tryptophan loading)	(102)
		(n.d.) 3-HK (urinary excretions following tryptophan loading)	
	Human	↓ KA (serum)	(103)
	Human	↑ QA (cerebrospinal fluid)	(104)
		↑ QA (cerebrospinal fluid, suicide attempters)	
		(n.d.) KA (cerebrospinal fluid, suicide attempters)	
	Human	↑ QA (cerebrospinal fluid, following suicide attempt)	(105)
		↓ KA (cerebrospinal fluid, following suicide attempt)	
	Human	(n.d.) KA (serum)	(106)
		(n.d.) QA (serum)	
	Human	(n.d.) KA (serum)	(107)
		(n.d.) 3-HK (serum)	
		(n.d.) QA (serum)	
	Human	↓ KA/QA ratio (serum)	(108)
		(n.d.) KA (serum)	
		(n.d.) 3-HK (serum)	
		(n.d.) QA (serum)	
Inflammation-associated depression	Human	↑ Kynurenine/KA ratio (interferon-α treatment, serum)	(109)
	Human	↓ KA (interferon-α treatment, serum)	(110)
	Human	↑ KA (interferon-α treatment, cerebrospinal fluid)	(12)
		↑ QA (interferon-α treatment, cerebrospinal fluid)	
		(n.d.) QA (interferon-α treatment, plasma)	
	C57BL/6J mice	↑ 3-HK [lipopolysaccharide (1 mg/kg), brain]	(23)
		↑ QA [lipopolysaccharide (1 mg/kg), brain]	
		(n.d.) KA [lipopolysaccharide (1 mg/kg), brain]	
Bipolar disorder (BPD)	Human	(n.d.) KA (post-mortem brain, anterior cingulate)	(111)
	Human	↓ KA (serum)	(112)
	Human	↑ KA (cerebrospinal fluid)	(113)
	Human	↑ KA (cerebrospinal fluid, with history of psychotic features)	(114)
	Human	↑ KA (cultured fibroblasts)	(115)
		↑ 3-HK (cultured fibroblasts)	
	Human	↑ KA [cerebrospinal fluid, with KMO Arg(452) mutant allele]	(80)
		↓ KMO expression [post-mortem brain, with KMO Arg(452) mutant allele, hippocampus]	
	Human	↓ KA/QA ratio (serum)	(116)
		(n.d.) KA (serum)	
		(n.d.) 3-HK (serum)	
		(n.d.) QA (serum)	
Schizophrenia (SCZ)	Human	(n.d.) QA (cerebrospinal fluid)	(83)
	Human	↑ KA (post-mortem brain, dorsolateral prefrontal cortex)	(117)
		(n.d.) KA (post-mortem brain, frontopolar area, or tertiary visual association cortex)	
		(n.d.) 3-HK (post-mortem brain, dorsolateral prefrontal cortex, frontopolar area, or tertiary visual association cortex)	
		
	Human	↑ KA (cerebrospinal fluid)	(118)
	Human	(n.d.) 3-HK (serum)	(119, 120)
	Human	↓ KA (serum)	(121)
		↑ 3-HK (serum)	
	Human	↑ KA (post-mortem brain, frontopolar area)	(122)
		(n.d.) KA (post-mortem brain, dorsolateral prefrontal cortex)	
		↓ KMO activity (post-mortem brain, dorsolateral prefrontal cortex, and frontopolar area)	
	Human	↑ KA (cerebrospinal fluid, with KMO rs1053230 single nucleotide polymorphism)	(79)
	Human	↑ KA (cerebrospinal fluid)	(123)
	Human	↓ KA (cerebrospinal fluid, following suicide attempt)	(124)
	Human	↑ KA (cultured fibroblasts)	(115)
		↑ 3-HK (cultured fibroblasts)	
	Human	↑ KA (cerebrospinal fluid)	(125)
		(n.d.) QA (cerebrospinal fluid)	
		↓ QA/KA ratio (cerebrospinal fluid)	
Neurodevelopmental disorders	Human	↑ KA (Down syndrome, post-mortem brain, frontal cortex)	(126)
		(n.d.) KA (Down syndrome, post-mortem brain, temporal cortex)	
	BTBR T+tf/J mice	KMO single nucleotide polymorphisms (3, autism spectrum disorder behavioral model)	(127)
	Human	(n.d.) KA (ADHD, serum)	(128)
		(n.d.) 3-HK (ADHD, serum)	

*Disruptions in kynurenine metabolites are described in Huntington’s disease (HD), Alzheimer’s disease (AD), depression, bipolar disorder (BPD), schizophrenia (SCZ), and neurodevelopmental disorders. Only three kynurenine metabolites, 3-HK, KA, and QA, were included in this table as they demonstrate potential changes in KMO activity that could result in neuropathological consequences. Only animal studies with endogenous model disease disruptions were incorporated in the table (i.e., genetic mutant model)*.

*(n.d.), no difference between disorder/mutant animal samples and healthy control/control animal samples*.

### Huntington’s disease

Huntington’s disease (HD) is a severe neurodegenerative disorder that is characterized by motor, cognitive, and neuropsychiatric symptoms. Early experimental models demonstrated that glutamate-mediated excitotoxicity in the basal ganglia could mirror the neuronal degeneration seen in HD patients (129). Therefore, it is possible that elevated levels of QA, which can cause excitotoxicity could contribute to the pathogenesis of HD. Though early attempts to measure QA in post-mortem cortical tissue indicated no difference between HD patients and controls, later studies demonstrated that both KA and 3-HK were elevated suggesting an overall increase in kynurenine metabolism (Table 1) (81, 84, 87, 88). More specifically, it was later established that both QA and 3-HK are increased in striatal and cortical regions during the early stages of HD (Grade 0/1), suggesting temporal specificity for the disruption in kynurenine metabolism (Table 1) (89). Further, HD patients present with elevated markers of oxidative stress, which can be propagated by 3-HK and QA supporting the hypothesis that these metabolites can contribute to HD pathogenesis (130, 131). The YAC128, R6/2, Hdh^Q92^/Hdh^Q111^, and FBV/N mutant HD mouse models all have endogenous disruptions in the kynurenine pathway that result an increase in neurotoxic metabolites that accumulate as the mice age (Table 1) (88, 90, 93). These mutant HD mouse models demonstrate that the development of HD-like pathology can parallel changes in kynurenine metabolism. As the enzyme upstream of the synthesis of both 3-HK and QA, KMO is a relevant potential therapeutic target for the modulation of this imbalance in kynurenine metabolites. Interestingly, inhibition of KMO ameliorates HD-associated neurodegeneration both in a *Drosophila melanogaster* HD fly model (Htt93Q) and in the R6/2 mutant HD mouse model (92, 100). Taken together, the data from patient analyses and preclinical studies demonstrate that HD is associated with elevated neurotoxic kynurenine metabolites, 3-HK and QA, and targeting KMO to repair this disease-associated imbalance has therapeutic promise in treating neurodegeneration.

Huntington’s disease is also considered to be an inflammatory disease as elevations in plasma inflammatory markers have been described both in patients and in a HD-mutant mouse model (132). This peripheral response most likely is a mirror of the neuroinflammatory response accompanying disease progression (133, 134). Neuroinflammation in HD has mainly been characterized by microglia activation particularly associated with the brain regions of greatest neurodegeneration (135, 136). Indeed, an imaging study demonstrated that in HD pre-symptomatic gene carriers, microglia activation was already elevated and it correlated with regions undergoing neurodegeneration (137). The R6/2 mutant HD mouse model also exhibits neuroinflammation and microglia activation associated with HD-like pathology development (138). Microglia are likely involved in the pathogenesis of HD and produce neurotoxic 3-HK and QA when activated providing a clear link between disrupted kynurenine metabolism and HD-associated pathology development.

### Alzheimer’s disease

Alzheimer’s disease (AD) is the most common form of dementia and is a serious neurodegenerative disorder defined by a dramatic decline in cognitive function. Though AD pathology is characterized by amyloid-β plaque and hyper-phosphorylated tau neurofibrillary tangle accumulation, the pathogenesis of this disease is still not fully understood (139). Early analysis of post-mortem brain samples from AD patients failed to reveal an increase in kynurenine metabolism (Table 1) (86, 87, 95). Even so, more recent assessments of peripheral kynurenine metabolites in AD patients have indicated that serum 3-HK and QA concentrations were elevated while the KA concentration decreased (Table 1) (98, 99, 101). Further, one study specifically demonstrated that serum QA concentration was inversely correlated with cognitive performance in AD patients (99). Kynurenine pathway activation and production of neurotoxic metabolites have also been characterized in association with AD pathology. Both IDO1 and QA were increased in AD post-mortem hippocampal tissue specifically associated with the perimeter of amyloid-β plaques (140). As the accumulation of plaques and tangles results in microglial activation and neuroinflammation, it is feasible that this stimulates kynurenine metabolism and neurotoxic metabolite production in AD (141). *In vitro*, Aβ-42, the neurotoxic constituent of amyloid-β plaques, can induce IDO1 upregulation and QA production in human macrophages and microglia (142). Elevated neurotoxic kynurenine metabolite production arising from microglia activation may in turn contribute to the pathogenesis of AD. As in HD, increased extracellular glutamate and neuronal excitotoxicity are thought to contribute to the degeneration of neurons as AD pathology accumulates (143, 144). AD patients also have elevated markers of oxidative stress, both of which an elevation in neurotoxic QA and 3-HK can propagate (145). Specifically, QA has been shown to prompt tau phosphorylation *in vitro* indicating that it may have a direct influence on development of AD-associated pathology (146). These data provide evidence that inflammation associated with markers of AD pathology may upregulate kynurenine metabolism and the production of neurotoxic metabolites. Though directly focusing on inflammation therapeutically has had little success in AD patients, targeting the kynurenine pathway, further downstream still remains a viable option. In the APPtg AD mouse model, peripheral inhibition of KMO attenuated spatial memory loss and synapse loss associated with an increase in brain KA (100). These data demonstrate that KMO could provide a treatment focus in AD with the intent of preventing neurotoxic metabolite accumulation.

Alzheimer’s disease is associated with the development and propagation of neuroinflammation in response to the accumulation of amyloid-β plaques and tau tangles. Post-mortem analyses have revealed that both microglia and astrocytes become upregulated and activated as AD-associated neuropathology develops (141, 147). One imaging study demonstrated that microglia activation is specifically correlated with cognitive status, which could not be predicted by amyloid-β load (148). Transgenic mouse models that develop AD-related neuropathology also exhibit microgliosis and astrocytosis associated with the advancement of pathology (149, 150). These data further demonstrate the significance of microglia to AD pathology and the relevance of neurotoxic kynurenine metabolites to the progression of AD.

### Depression

Depression is a highly debilitating neuropsychiatric disorder with a diverse symptom profile, including depressed mood, anxiety, anhedonia, fatigue, and cognitive impairment. Unfortunately, less than half of affected individuals experience satisfactory therapeutic benefit from typical antidepressant treatments (151). Studies describing kynurenine metabolism in patients with depression report increases in CSF QA and decreases in plasma KA and the KA/QA ratio (Table 1) (103, 104, 108). Despite these data, other studies report no difference in kynurenine metabolism between patients with depression and controls (Table 1) (106, 107). Analysis of kynurenine metabolites in patients with depression who had also attempted suicide revealed an elevation in CSF QA and a decrease in KA (Table 1) (104, 105). These results suggest a role for altered kynurenine metabolism in the pathogenesis of depression, and additional studies have demonstrated that inflammation can induce both depression symptoms and metabolic changes. Specifically, interferon-α therapy, used to treat hepatitis C, increases the prevalence of depression symptoms in the patients receiving treatment, and the severity of symptoms are correlated with elevations in CSF QA (12, 109). It was recently proposed that kynurenine pathway enzyme expression could be a valid etiological classification system for patients diagnosed with depression (152). As inflammation-induced alterations in the kynurenine pathway represents a significant gene by environment interaction, such a classification system could potentially provide a more precise way to treat patients, both genetically and pharmacologically.

Inflammation is hypothesized to be a major contributor to the pathogenesis of depression and clinically, administration of endotoxin can directly precipitate depression symptoms (153, 154). Further, patients who suffer from chronic inflammatory diseases are significantly more likely to develop symptoms of depression than healthy adults (155). While direct clinical evidence in patients of depression that inflammation activates microglia and upregulates KMO within the brain is lacking, it is feasible that inflammation induces an imbalance in kynurenine metabolism elevating neurotoxic QA production that results in depression-related symptoms (10, 156). Preclinically, inflammatory stimuli induce depressive-like behaviors that are dependent on increased kynurenine production by IDO1, demonstrating that kynurenine metabolism is necessary for inflammation-related behaviors (11, 157–160). Our lab recently demonstrated that KMO-deficient mice were protected from inflammation-induced deficits in recognition memory, a depressive-like behavior (160). These data provide preliminary evidence that inflammation-induced depressive-like behaviors are specifically dependent on neurotoxic kynurenine metabolism. A recent study demonstrated that lipopolysaccharide (LPS)-induced depressive-like behaviors can be attenuated with administration of the NMDAR antagonist ketamine, and LPS treatment induces accumulation of central 3-HK and QA (23). These results indicate that inflammation-induced depressive-like behavioral changes are in part mediated by NMDAR activation, which could potentially be accomplished by the elevation in QA, an NMDAR agonist. Inflammatory stimuli can undoubtedly alter kynurenine metabolism favoring neurotoxic metabolism through KMO, which appears to contribute to the mechanism by which inflammation results in depression and related behaviors.

Though post-mortem samples from patients with depression have been analyzed for microglia activation, the results from multiple studies are inconsistent (161, 162). They generally favor elevated microglia activation in patients with depression and a significant increase in activation in patients who also committed suicide (163). Preclinically, inflammatory stimuli can induce depressive-like behaviors that are associated with a neuroinflammatory response including microglia activation (164). Minocycline, a microglial activation inhibitor, has been demonstrated to attenuate inflammation-induced depressive-like behaviors (165). Together, these data strongly suggest that microglia could potentially be involved in the pathogenesis of depression behaviors that are associated with inflammation and altered kynurenine metabolism.

### Bipolar disorder

BPD is defined by alternating periods of mania, which often features psychosis, and depression interspersed with symptom-free intervals, known as euthymia (166). Further, cognitive impairments such as those in executive functioning and verbal memory are present in all states of BPD (167). During the euthymic phase of patients with BPD, the concentration of CSF KA was found to be significantly elevated (Table 1) (113). Specifically, higher levels of CSF KA were correlated with a history of psychosis or having experienced a recent manic episode in BPD patients (Table 1) (114). Acute psychotic symptoms in HIV-1-infected patients were also associated with elevations in CSF KA, further confirming the significance of alterations in KA concentration on the development of psychosis behavior (168). Dysregulation in the DA system is thought to be one of the mechanisms responsible for BPD symptomology, therefore it is possible that KA influences psychosis through its interaction with DA (169, 170). Analysis of the KMO Arg(452) mutant allele in BPD patients, which reduces KMO expression and increases KA production, also revealed an association with psychotic symptoms during manic episodes (Table 1) (80). These are further data to support the role of KMO in the production of KA and the influence of KA concentration on psychosis in BPD.

Another mechanism thought to contribute to the pathogenesis of BPD is immune activation, specifically through the production of pro-inflammatory cytokines (171, 172). Recent data points to an explicit role for IL-1β as CSF concentrations were increased in euthymic BPD patients compared to controls and were correlated with a recent history of at least one manic episode (173). Associated with this inflammatory paradigm, a recent study found that the serum KA/QA ratio was decreased in BPD patients contrary to previous studies demonstrating an elevation in KA (Table 1) (116). Further, isolation of BPD patient skin fibroblasts demonstrated increased baseline production of both KA and 3-HK, however, pro-inflammatory cytokine stimulation only resulted in elevated 3-HK accumulation (115). In accord with this hypothesis, elevated neuroinflammatory markers and glia cell (microglia and astrocytes) activation have been described in post-mortem BPD patient tissue samples (174, 175). Neuroinflammation in BPD patients would propagate the production of neurotoxic kynurenine metabolites similar to those that have already been described in patients. Though studies may support alternate activation of these two branches of metabolism in BPD, it is clear that there is a disruption that could be therapeutically targeted by focusing on altering kynurenine metabolism at KMO in microglia.

### Schizophrenia

SCZ is a neuropsychiatric disorder defined by positive symptoms (psychosis and paranoia), negative symptoms (social deficits and anhedonia), and cognitive dysfunction. The mechanisms underlying the pathogenesis of SCZ are not well understood. Currently, it is believed that SCZ results from neurocircuit-level disruptions in neurotransmitters including dysfunction in DA and glutamate systems (176). Post-mortem tissue analysis of SCZ patients revealed an increase in KA that was associated with decreased KMO activity rather than alterations in KAT activity (Table 1) (117, 122). CSF KA concentration was also elevated in SCZ patients while there was no change in CSF QA concentration (Table 1) (118, 123, 125). Interestingly, CSF KA was significantly decreased in SCZ patients who attempted suicide compared to non-attempters (Table 1) (124). Studies that were conducted to determine the correlation between KMO gene polymorphisms and SCZ diagnosis did not reveal any significant relationships (77, 177). However, one KMO polymorphism that potentially impacts substrate interaction, rs1053230, correlated with elevated CSF KA concentrations in SCZ patients, further demonstrating the link between KMO and disrupted kynurenine metabolism (Table 1) (79). Studies have demonstrated that KA can disrupt cognitive performance through its interaction with the glutamate system and it has the potential to contribute to psychotic symptoms by influencing DA signaling. Preclinically, reducing endogenous levels of KA can prevent amphetamine- and ketamine-induced disruptions in sustained attention, working memory, and spatial memory tasks, paradigms used to experimentally model SCZ deficits (178). Together, these data provide justification for better understanding the impact of disrupted kynurenine metabolism on SCZ pathogenesis and the potential relevance of targeting KMO therapeutically in SCZ.

While the potential for the involvement of inflammation and pro-inflammatory cytokines in the pathogenesis of SCZ exists, the data analyzing the supporting neuropathological evidence are inconsistent. Initial examination of SCZ post-mortem tissue indicated a reduction in density of astrocyte markers (GFAP) when compared to normal controls (179). More recent SCZ post-mortem tissue investigations have revealed upregulation of both microglia and astrocyte markers (180–182). An additional binding analysis revealed that QA-specific microglia were reduced in SCZ, but overall microglia density was unchanged (183). A recent imaging study in SCZ patients found no difference in microglia activation in white or gray matter, contradicting the multiple post-mortem studies (184). Despite these varying results, it is clear that there is potential for a pathological role of neuroinflammation and glia involvement in SCZ (185).

### Neurodevelopment

Brain development and maturation is a dynamic process that begins in the embryo and continues well into the young adult years (186). However, neurodevelopment appears to be quite susceptible to disruptions in kynurenine metabolism. Kynurenine supplemented chow fed to gestating mothers prenatally and offspring postnatally elevates KA in adolescences and adults (187, 188). Exogenous kynurenine treatment during these periods also results in disruptions in contextual memory, spatial working memory, and cognitive flexibility in adults (187–190). Specifically, elevating KA with kynurenine during a prenatal period decreases dendritic spine density and markers of excitatory transmission (evoked glutamate release and mGluR2 receptor expression) (190). Kynurenine metabolism during neurodevelopment was also targeted by administering a KMO inhibitor to pregnant dams in late gestation, which results in an immediate increase in KA in the embryo (191). In adolescence, this treatment results in an increase in neuronal excitability, an increase in GluN2A/2B subunits of the NMDAR and elevated PSD95, a post-synaptic density marker (191). Adults also show changes in excitability, decreases in dendritic complexity and number, disruptions in long-term potentiation, and increased neurogenesis (192–194). These results clearly demonstrate that alterations in kynurenine metabolism occurring during development can significantly impact normal neuronal functioning in adolescence and continue into adulthood. The BTBR T+tf/J spontaneous mutant mouse has been used preclinically to model behaviors of autism spectrum disorder (ASD), one neurodevelopmental disorder (195). The BTBR mouse model is characterized by repetitive behaviors, communication deficits, and social deficits that are also associated with three KMO polymorphisms specific to the BTBR strain (127). This correlation suggests that in the BTBR mouse model, a disruption in KMO function may contribute to the development of ASD-like behavior. Though there are only a couple of studies analyzing kynurenine metabolite disruptions in the context of neurodevelopmental disorders (see Table 1), the strong preclinical data supports the importance of KMO and its regulation in proper development.

Immune activation, either in pregnant mothers or in early development, provides a more clinically relevant model for determining the impact of disrupted kynurenine metabolism during neurodevelopment. Preclinical maternal immune activation (MIA) is modeled by administration of Poly I:C, an immune mimetic, to pregnant dams, which results in disruptions in sensorimotor gating and spontaneous locomotor activity in adult offspring (196, 197). There are also significant increases in microglia activation in the offspring of MIA dams (197, 198). When neonatal pups are given an immune stimulus (Poly I:C, influenza, LPS), this also results in disturbances in adulthood including impaired sensorimotor gating and working memory concurrent with elevated microglia activation (199–201). Though these models demonstrate the association of microglia activation with behaviors that result from impaired neurodevelopment, few studies have directly characterized microglia activation in patients with neurodevelopmental disorders. A recent imaging study established that young adults diagnosed with ASD have significantly elevated microglia activation in multiple brain regions (202). These data further support the possibility that microglia contribute to neurodevelopmental disorders, both during development and throughout maturation.

## Conclusion/Future Research

Kynurenine metabolites have the capacity to directly or indirectly affect neuronal functioning under both basal and during ­pathological conditions. Though evidence suggests that multiple neurological disorders are associated with disruptions in central kynurenine metabolism (Table 1), it is unclear whether these changes occur during disease progression or contribute directly to disease initiation. Determination of the specific impact that disruptions in kynurenine metabolism have on the pathogenesis of neurological disorders will allow for better understanding of individual diseases and provide an additional therapeutic objective. Preclinical evidence demonstrates that neurotoxic QA and inhibitory KA can both be detrimental physiologically and behaviorally when levels become elevated. Therefore, with the capacity to direct kynurenine metabolism to both QA and KA, KMO provides a potential target for treatment intervention. While the ability to modulate KMO activity would be a powerful tool to determine if targeting it therapeutically is feasible, few techniques currently exist with therapeutic potential. Until recently, KMO pharmacological inhibitors were limited modulating kynurenine metabolism to the periphery as they did not cross the blood–brain barrier (9). However, a newly described competitive inhibitor was demonstrated preclinically to have the ability to inhibit KMO peripherally and partially penetrate the blood–brain barrier (203). Though proposed for therapeutic use in HD patients, the data reviewed here clearly demonstrate that treatment with an effective KMO inhibitor could potentially be beneficial to patients diagnosed with other neurological disorders. Additionally, KMO-deficient mice have biochemically characterized providing a valuable preclinical research tool to further explore the biological and pathophysiological role of KMO (204). Whether disrupted kynurenine metabolism is a biomarker of disease risk or a treatment target for slowing the disease progression, it is clear that the kynurenine pathway is clinically relevant to neurological disorders. Therefore, it is necessary to understand the significance of maintaining the balance between KA and QA production through KMO, both in health and neurological diseases.

## Conflict of Interest Statement

Dr. Jason C. O’Connor has received funding from Janssen Research and Development, LLC for work that is not related to the material presented or discussed in the article submitted for consideration. Jennifer M. Parrott declares that the research was conducted in the absence of any commercial or financial relationships that could be construed as a potential conflict of interest.

## Funding

JP: National Institute of Mental Health: 1F31MH102070-01A1. JOC: National Institute of Mental Health: MH090127, National Center for Advancing Translational Studies: UL1TR001120, Texas Higher Education Coordinating Board: 003659-0010-2013.

## References

[1] Beadle GW Mitchell HK Nyc JF Kynurenine as an intermediate in the formation of nicotinic acid from tryptophane by neurospora. Proc Natl Acad Sci U S A (1947) 33:155–8. 10.1073/pnas.33.6.155 16588735 PMC1079015

[2] Rubin RT Multiple biochemical correlates of manic-depressive illness. J Psychosom Res (1968) 12:171–80. 10.1016/0022-3999(68)90025-1 5685300

[3] Curzon G Bridges PK. Tryptophan metabolism in depression. J Neurol Neurosurg Psychiatry (1970) 33:698–704. 10.1136/jnnp.33.5.698 5478953 PMC493552

[4] Ganong AH Cotman CW. Kynurenic acid and quinolinic acid act at N-methyl-d-aspartate receptors in the rat hippocampus. J Pharmacol Exp Ther (1986) 236:293–9. 2867215

[5] Heyes MP Saito K Crowley JS Davis LE Demitrack MA Der M Quinolinic acid and kynurenine pathway metabolism in inflammatory and non-inflammatory neurological disease. Brain (1992) 115(Pt 5):1249–73. 10.1093/brain/115.5.1249 1422788

[6] Danesch U Hashimoto S Renkawitz R Schutz G. Transcriptional regulation of the tryptophan oxygenase gene in rat liver by glucocorticoids. J Biol Chem (1983) 258:4750–3. 6187742

[7] Saito K Lackner A Markey SP Heyes MP. Cerebral cortex and lung indoleamine-2,3-dioxygenase activity is increased in type-D retrovirus infected macaques. Brain Res (1991) 540:353–6. 10.1016/0006-8993(91)90536-5 1647247

[8] Gal EM Sherman AD. l-kynurenine: its synthesis and possible regulatory function in brain. Neurochem Res (1980) 5:223–39. 10.1007/BF00964611 6154900

[9] Schwarcz R Bruno JP Muchowski PJ Wu HQ. Kynurenines in the mammalian brain: when physiology meets pathology. Nat Rev Neurosci (2012) 13:465–77. 10.1038/nrn3257 22678511 PMC3681811

[10] Dantzer R O’Connor JC Lawson MA Kelley KW. Inflammation-associated depression: from serotonin to kynurenine. Psychoneuroendocrinology (2011) 36:426–36. 10.1016/j.psyneuen.2010.09.012 21041030 PMC3053088

[11] O’Connor JC Lawson MA Andre C Moreau M Lestage J Castanon N Lipopolysaccharide-induced depressive-like behavior is mediated by indoleamine 2,3-dioxygenase activation in mice. Mol Psychiatry (2009) 14:511–22. 10.1038/sj.mp.4002148 18195714 PMC2683474

[12] Raison CL Dantzer R Kelley KW Lawson MA Woolwine BJ Vogt G CSF concentrations of brain tryptophan and kynurenines during immune stimulation with IFN-alpha: relationship to CNS immune responses and depression. Mol Psychiatry (2010) 15:393–403. 10.1038/mp.2009.116 19918244 PMC2844942

[13] Wang Y Liu H Mckenzie G Witting PK Stasch JP Hahn M Kynurenine is an endothelium-derived relaxing factor produced during inflammation. Nat Med (2010) 16:279–85. 10.1038/nm.2092 20190767 PMC3556275

[14] Opitz CA Litzenburger UM Sahm F Ott M Tritschler I Trump S An endogenous tumour-promoting ligand of the human aryl hydrocarbon receptor. Nature (2011) 478:197–203. 10.1038/nature10491 21976023

[15] Hilmas C Pereira EF Alkondon M Rassoulpour A Schwarcz R Albuquerque EX. The brain metabolite kynurenic acid inhibits alpha7 nicotinic receptor activity and increases non-alpha7 nicotinic receptor expression: physiopathological implications. J Neurosci (2001) 21:7463–73. 11567036 10.1523/JNEUROSCI.21-19-07463.2001 PMC6762893

[16] Kiss C Ceresoli-Borroni G Guidetti P Zielke CL Zielke HR Schwarcz R. Kynurenate production by cultured human astrocytes. J Neural Transm (2003) 110:1–14. 10.1007/s00702-002-0770-z 12541009

[17] Rossi F Schwarcz R Rizzi M. Curiosity to kill the KAT (kynurenine aminotransferase): structural insights into brain kynurenic acid synthesis. Curr Opin Struct Biol (2008) 18:748–55. 10.1016/j.sbi.2008.09.009 18950711

[18] Foster AC Vezzani A French ED Schwarcz R. Kynurenic acid blocks neurotoxicity and seizures induced in rats by the related brain metabolite quinolinic acid. Neurosci Lett (1984) 48:273–8. 10.1016/0304-3940(84)90050-8 6237279

[19] Erhardt S Schwieler L Emanuelsson C Geyer M. Endogenous kynurenic acid disrupts prepulse inhibition. Biol Psychiatry (2004) 56:255–60. 10.1016/j.biopsych.2004.06.006 15312813

[20] Chess AC Bucci DJ. Increased concentration of cerebral kynurenic acid alters stimulus processing and conditioned responding. Behav Brain Res (2006) 170:326–32. 10.1016/j.bbr.2006.03.006 16621049

[21] Chess AC Simoni MK Alling TE Bucci DJ. Elevations of endogenous kynurenic acid produce spatial working memory deficits. Schizophr Bull (2007) 33:797–804. 10.1093/schbul/sbl033 16920787 PMC2526148

[22] Guillemin GJ Smythe G Takikawa O Brew BJ. Expression of indoleamine 2,3-dioxygenase and production of quinolinic acid by human microglia, astrocytes, and neurons. Glia (2005) 49:15–23. 10.1002/glia.20090 15390107

[23] Walker AK Budac DP Bisulco S Lee AW Smith RA Beenders B NMDA receptor blockade by ketamine abrogates lipopolysaccharide-induced depressive-like behavior in C57BL/6J mice. Neuropsychopharmacology (2013) 38:1609–16. 10.1038/npp.2013.71 23511700 PMC3717543

[24] Eastman CL Guilarte TR. Cytotoxicity of 3-hydroxykynurenine in a neuronal hybrid cell line. Brain Res (1989) 495:225–31. 10.1016/0006-8993(89)90216-3 2765927

[25] Eastman CL Guilarte TR. The role of hydrogen peroxide in the in vitro cytotoxicity of 3-hydroxykynurenine. Neurochem Res (1990) 15:1101–7. 10.1007/BF01101711 2089269

[26] Okuda S Nishiyama N Saito H Katsuki H. Hydrogen peroxide-mediated neuronal cell death induced by an endogenous neurotoxin, 3-hydroxykynurenine. Proc Natl Acad Sci U S A (1996) 93:12553–8. 10.1073/pnas.93.22.12553 8901620 PMC38030

[27] Colin-Gonzalez AL Maldonado PD Santamaria A. 3-Hydroxykynurenine: an intriguing molecule exerting dual actions in the central nervous system. Neurotoxicology (2013) 34:189–204. 10.1016/j.neuro.2012.11.007 23219925

[28] Lee SM Lee YS Choi JH Park SG Choi IW Joo YD Tryptophan metabolite 3-hydroxyanthranilic acid selectively induces activated T cell death via intracellular GSH depletion. Immunol Lett (2010) 132:53–60. 10.1016/j.imlet.2010.05.008 20570696

[29] Lee WS Lee SM Kim MK Park SG Choi IW Choi I The tryptophan metabolite 3-hydroxyanthranilic acid suppresses T cell responses by inhibiting dendritic cell activation. Int Immunopharmacol (2013) 17:721–6. 10.1016/j.intimp.2013.08.018 24029595

[30] Morita T Saito K Takemura M Maekawa N Fujigaki S Fujii H l-tryptophan-kynurenine pathway metabolite 3-hydroxyanthranilic acid induces apoptosis in macrophage-derived cells under pathophysiological conditions. Adv Exp Med Biol (1999) 467:559–63. 10.1007/978-1-4615-4709-9_69 10721100

[31] Morita T Saito K Takemura M Maekawa N Fujigaki S Fujii H 3-Hydroxyanthranilic acid, an l-tryptophan metabolite, induces apoptosis in monocyte-derived cells stimulated by interferon-gamma. Ann Clin Biochem (2001) 38:242–51. 10.1258/0004563011900461 11392499

[32] Krause D Suh HS Tarassishin L Cui QL Durafourt BA Choi N The tryptophan metabolite 3-hydroxyanthranilic acid plays anti-inflammatory and neuroprotective roles during inflammation: role of hemeoxygenase-1. Am J Pathol (2011) 179:1360–72. 10.1016/j.ajpath.2011.05.048 21855684 PMC3157215

[33] Stone TW Perkins MN Quinolinic acid: a potent endogenous excitant at amino acid receptors in CNS. Eur J Pharmacol (1981) 72:411–2. 10.1016/0014-2999(81)90587-2 6268428

[34] Lapin IP. Stimulant and convulsive effects of kynurenines injected into brain ventricles in mice. J Neural Transm (1978) 42:37–43. 10.1007/BF01262727 641543

[35] Schwarcz R Whetsell WO Jr Mangano RM. Quinolinic acid: an endogenous metabolite that produces axon-sparing lesions in rat brain. Science (1983) 219:316–8. 10.1126/science.6849138 6849138

[36] Rios C Santamaria A. Quinolinic acid is a potent lipid peroxidant in rat brain homogenates. Neurochem Res (1991) 16:1139–43. 10.1007/BF00966592 1686636

[37] Stone TW Behan WM Macdonald M Darlington LG. Possible mediation of quinolinic acid-induced hippocampal damage by reactive oxygen species. Amino Acids (2000) 19:275–81. 10.1007/s007260070059 11026499

[38] Lugo-Huitron R Ugalde Muniz P Pineda B Pedraza-Chaverri J Rios C Perez-De La Cruz V. Quinolinic acid: an endogenous neurotoxin with multiple targets. Oxid Med Cell Longev (2013) 2013:104024. 10.1155/2013/104024 24089628 PMC3780648

[39] Phillips RS. Structure, mechanism, and substrate specificity of kynureninase. Biochim Biophys Acta (2011) 1814:1481–8. 10.1016/j.bbapap.2010.12.003 21167323 PMC3102132

[40] Baran H Schwarcz R. Presence of 3-hydroxyanthranilic acid in rat tissues and evidence for its production from anthranilic acid in the brain. J Neurochem (1990) 55:738–44. 10.1111/j.1471-4159.1990.tb04553.x 2384749

[41] Gobaille S Kemmel V Brumaru D Dugave C Aunis D Maitre M. Xanthurenic acid distribution, transport, accumulation and release in the rat brain. J Neurochem (2008) 105:982–93. 10.1111/j.1471-4159.2008.05219.x 18182052

[42] Copeland CS Neale SA Salt TE. Actions of xanthurenic acid, a putative endogenous group II metabotropic glutamate receptor agonist, on sensory transmission in the thalamus. Neuropharmacology (2013) 66:133–42. 10.1016/j.neuropharm.2012.03.009 22491023

[43] Neale SA Copeland CS Uebele VN Thomson FJ Salt TE. Modulation of hippocampal synaptic transmission by the kynurenine pathway member xanthurenic acid and other VGLUT inhibitors. Neuropsychopharmacology (2013) 38:1060–7. 10.1038/npp.2013.4 23303071 PMC3629405

[44] Dykens JA Sullivan SG Stern A. Oxidative reactivity of the tryptophan metabolites 3-hydroxyanthranilate, cinnabarinate, quinolinate and picolinate. Biochem Pharmacol (1987) 36:211–7. 10.1016/0006-2952(87)90691-5 2949752

[45] Christen S Southwell-Keely PT Stocker R. Oxidation of 3-hydroxyanthranilic acid to the phenoxazinone cinnabarinic acid by peroxyl radicals and by compound I of peroxidases or catalase. Biochemistry (1992) 31:8090–7. 10.1021/bi00149a045 1324727

[46] Fazio F Lionetto L Molinaro G Bertrand HO Acher F Ngomba RT Cinnabarinic acid, an endogenous metabolite of the kynurenine pathway, activates type 4 metabotropic glutamate receptors. Mol Pharmacol (2012) 81:643–56. 10.1124/mol.111.074765 22311707

[47] Pucci L Perozzi S Cimadamore F Orsomando G Raffaelli N. Tissue expression and biochemical characterization of human 2-amino 3-carboxymuconate 6-semialdehyde decarboxylase, a key enzyme in tryptophan catabolism. FEBS J (2007) 274:827–40. 10.1111/j.1742-4658.2007.05635.x 17288562

[48] Kalisch BE Jhamandas K Boegman RJ Beninger RJ. Picolinic acid protects against quinolinic acid-induced depletion of NADPH diaphorase containing neurons in the rat striatum. Brain Res (1994) 668:1–8. 10.1016/0006-8993(94)90504-5 7535651

[49] Guidetti P Hoffman GE Melendez-Ferro M Albuquerque EX Schwarcz R. Astrocytic localization of kynurenine aminotransferase II in the rat brain visualized by immunocytochemistry. Glia (2007) 55:78–92. 10.1002/glia.20432 17024659

[50] Braidy N Grant R Adams S Brew BJ Guillemin GJ. Mechanism for quinolinic acid cytotoxicity in human astrocytes and neurons. Neurotox Res (2009) 16:77–86. 10.1007/s12640-009-9051-z 19526301

[51] Saito K Markey SP Heyes MP. Effects of immune activation on quinolinic acid and neuroactive kynurenines in the mouse. Neuroscience (1992) 51:25–39. 10.1016/0306-4522(92)90467-G 1465184

[52] Irwin MR Cole SW. Reciprocal regulation of the neural and innate immune systems. Nat Rev Immunol (2011) 11:625–32. 10.1038/nri3042 21818124 PMC3597082

[53] Saijo K Glass CK. Microglial cell origin and phenotypes in health and disease. Nat Rev Immunol (2011) 11:775–87. 10.1038/nri3086 22025055

[54] Di Filippo M Sarchielli P Picconi B Calabresi P. Neuroinflammation and synaptic plasticity: theoretical basis for a novel, immune-centred, therapeutic approach to neurological disorders. Trends Pharmacol Sci (2008) 29:402–12. 10.1016/j.tips.2008.06.005 18617277

[55] Nakagami Y Saito H Katsuki H. 3-Hydroxykynurenine toxicity on the rat striatum in vivo. Jpn J Pharmacol (1996) 71:183–6. 10.1254/jjp.71.183 8835646

[56] Christen S Peterhans E Stocker R. Antioxidant activities of some tryptophan metabolites: possible implication for inflammatory diseases. Proc Natl Acad Sci U S A (1990) 87:2506–10. 10.1073/pnas.87.7.2506 2320571 PMC53718

[57] Leipnitz G Schumacher C Dalcin KB Scussiato K Solano A Funchal C In vitro evidence for an antioxidant role of 3-hydroxykynurenine and 3-hydroxyanthranilic acid in the brain. Neurochem Int (2007) 50:83–94. 10.1016/j.neuint.2006.04.017 16959377

[58] Reyes Ocampo J Lugo Huitron R Gonzalez-Esquivel D Ugalde-Muniz P Jimenez-Anguiano A Pineda B Kynurenines with neuroactive and redox properties: relevance to aging and brain diseases. Oxid Med Cell Longev (2014) 2014:646909. 10.1155/2014/646909 24693337 PMC3945746

[59] Santamaria A Rios C. MK-801, an N-methyl-d-aspartate receptor antagonist, blocks quinolinic acid-induced lipid peroxidation in rat corpus striatum. Neurosci Lett (1993) 159:51–4. 10.1016/0304-3940(93)90796-N 8264978

[60] Behan WM Mcdonald M Darlington LG Stone TW. Oxidative stress as a mechanism for quinolinic acid-induced hippocampal damage: protection by melatonin and deprenyl. Br J Pharmacol (1999) 128:1754–60. 10.1038/sj.bjp.0702940 10588931 PMC1571800

[61] Rodriguez-Martinez E Camacho A Maldonado PD Pedraza-Chaverri J Santamaria D Galvan-Arzate S Effect of quinolinic acid on endogenous antioxidants in rat corpus striatum. Brain Res (2000) 858:436–9. 10.1016/S0006-8993(99)02474-9 10708698

[62] Heyes MP Achim CL Wiley CA Major EO Saito K Markey SP. Human microglia convert l-tryptophan into the neurotoxin quinolinic acid. Biochem J (1996) 320(Pt 2):595–7. 8973572 10.1042/bj3200595 PMC1217971

[63] Foster AC Zinkand WC Schwarcz R. Quinolinic acid phosphoribosyltransferase in rat brain. J Neurochem (1985) 44:446–54. 10.1111/j.1471-4159.1985.tb05435.x 2578178

[64] Foster AC White RJ Schwarcz R. Synthesis of quinolinic acid by 3-hydroxyanthranilic acid oxygenase in rat brain tissue in vitro. J Neurochem (1986) 47:23–30. 10.1111/j.1471-4159.1986.tb02826.x 2940338

[65] Tavares RG Tasca CI Santos CE Alves LB Porciuncula LO Emanuelli T Quinolinic acid stimulates synaptosomal glutamate release and inhibits glutamate uptake into astrocytes. Neurochem Int (2002) 40:621–7. 10.1016/S0197-0186(01)00133-4 11900857

[66] Sattler R Tymianski M. Molecular mechanisms of glutamate receptor-mediated excitotoxic neuronal cell death. Mol Neurobiol (2001) 24(1–3):107–29. 10.1385/MN 11831548

[67] Tavares RG Schmidt AP Abud J Tasca CI Souza DO. In vivo quinolinic acid increases synaptosomal glutamate release in rats: reversal by guanosine. Neurochem Res (2005) 30:439–44. 10.1007/s11064-005-2678-0 16076013

[68] Beninger RJ Jhamandas K Boegman RJ El-Defrawy SR. Kynurenic acid-induced protection of neurochemical and behavioural deficits produced by quinolinic acid injections into the nucleus basalis of rats. Neurosci Lett (1986) 68:317–21. 10.1016/0304-3940(86)90509-4 2944036

[69] Stone TW. Neuropharmacology of quinolinic and kynurenic acids. Pharmacol Rev (1993) 45:309–79. 8248282

[70] Erhardt S Engberg G. Increased phasic activity of dopaminergic neurones in the rat ventral tegmental area following pharmacologically elevated levels of endogenous kynurenic acid. Acta Physiol Scand (2002) 175:45–53. 10.1046/j.1365-201X.2002.00962.x 11982504

[71] Nilsson LK Linderholm KR Erhardt S. Subchronic treatment with kynurenine and probenecid: effects on prepulse inhibition and firing of midbrain dopamine neurons. J Neural Transm (2006) 113:557–71. 10.1007/s00702-005-0343-z 16082514

[72] Linderholm KR Andersson A Olsson S Olsson E Snodgrass R Engberg G Activation of rat ventral tegmental area dopamine neurons by endogenous kynurenic acid: a pharmacological analysis. Neuropharmacology (2007) 53:918–24. 10.1016/j.neuropharm.2007.09.003 17959203

[73] Lopes C Pereira EF Wu HQ Purushottamachar P Njar V Schwarcz R Competitive antagonism between the nicotinic allosteric potentiating ligand galantamine and kynurenic acid at alpha7* nicotinic receptors. J Pharmacol Exp Ther (2007) 322:48–58. 10.1124/jpet.107.123109 17446300

[74] Beggiato S Antonelli T Tomasini MC Tanganelli S Fuxe K Schwarcz R Kynurenic acid, by targeting alpha7 nicotinic acetylcholine receptors, modulates extracellular GABA levels in the rat striatum in vivo. Eur J Neurosci (2013) 37:1470–7. 10.1111/ejn.12160 23442092

[75] Chess AC Landers AM Bucci DJ. l-kynurenine treatment alters contextual fear conditioning and context discrimination but not cue-specific fear conditioning. Behav Brain Res (2009) 201:325–31. 10.1016/j.bbr.2009.03.013 19428652

[76] Craddock N O’Donovan MC Owen MJ. The genetics of schizophrenia and bipolar disorder: dissecting psychosis. J Med Genet (2005) 42:193–204. 10.1136/jmg.2005.030718 15744031 PMC1736023

[77] Holtze M Saetre P Erhardt S Schwieler L Werge T Hansen T Kynurenine 3-monooxygenase (KMO) polymorphisms in schizophrenia: an association study. Schizophr Res (2011) 127:270–2. 10.1016/j.schres.2010.10.002 21030213

[78] Wonodi I Stine OC Sathyasaikumar KV Roberts RC Mitchell BD Hong LE Downregulated kynurenine 3-monooxygenase gene expression and enzyme activity in schizophrenia and genetic association with schizophrenia endophenotypes. Arch Gen Psychiatry (2011) 68:665–74. 10.1001/archgenpsychiatry.2011.71 21727251 PMC3855543

[79] Holtze M Saetre P Engberg G Schwieler L Werge T Andreassen OA Kynurenine 3-monooxygenase polymorphisms: relevance for kynurenic acid synthesis in patients with schizophrenia and healthy controls. J Psychiatry Neurosci (2012) 37:53–7. 10.1503/jpn.100175 21693093 PMC3244499

[80] Lavebratt C Olsson S Backlund L Frisen L Sellgren C Priebe L The KMO allele encoding Arg452 is associated with psychotic features in bipolar disorder type 1, and with increased CSF KYNA level and reduced KMO expression. Mol Psychiatry (2014) 19:334–41. 10.1038/mp.2013.11 23459468 PMC4990004

[81] Connick JH Stone TW Carla V Moroni F Increased kynurenic acid levels in Huntington’s disease. Lancet (1988) 2:1373 10.1016/S0140-6736(88)90918-X 2904097

[82] Reynolds GP Pearson SJ Halket J Sandler M. Brain quinolinic acid in Huntington’s disease. J Neurochem (1988) 50:1959–60. 10.1111/j.1471-4159.1988.tb02503.x 2967352

[83] Schwarcz R Tamminga CA Kurlan R Shoulson I. Cerebrospinal fluid levels of quinolinic acid in Huntington’s disease and schizophrenia. Ann Neurol (1988) 24:580–2. 10.1002/ana.410240417 2977086

[84] Reynolds GP Pearson SJ Increased brain 3-hydroxykynurenine in Huntington’s disease. Lancet (1989) 2:979–80. 10.1016/S0140-6736(89)90987-2 2571888

[85] Beal MF Matson WR Swartz KJ Gamache PH Bird ED. Kynurenine pathway measurements in Huntington’s disease striatum: evidence for reduced formation of kynurenic acid. J Neurochem (1990) 55:1327–39. 10.1111/j.1471-4159.1990.tb03143.x 2144582

[86] Beal MF Matson WR Storey E Milbury P Ryan EA Ogawa T Kynurenic acid concentrations are reduced in Huntington’s disease cerebral cortex. J Neurol Sci (1992) 108:80–7. 10.1016/0022-510X(92)90191-M 1385624

[87] Pearson SJ Reynolds GP. Increased brain concentrations of a neurotoxin, 3-hydroxykynurenine, in Huntington’s disease. Neurosci Lett (1992) 144:199–201. 10.1016/0304-3940(92)90749-W 1436703

[88] Guidetti P Reddy PH Tagle DA Schwarcz R. Early kynurenergic impairment in Huntington’s disease and in a transgenic animal model. Neurosci Lett (2000) 283:233–5. 10.1016/S0304-3940(00)00956-3 10754231

[89] Guidetti P Luthi-Carter RE Augood SJ Schwarcz R. Neostriatal and cortical quinolinate levels are increased in early grade Huntington’s disease. Neurobiol Dis (2004) 17:455–61. 10.1016/j.nbd.2004.07.006 15571981

[90] Guidetti P Bates GP Graham RK Hayden MR Leavitt BR Macdonald ME Elevated brain 3-hydroxykynurenine and quinolinate levels in Huntington disease mice. Neurobiol Dis (2006) 23:190–7. 10.1016/j.nbd.2006.02.011 16697652

[91] Sathyasaikumar KV Stachowski EK Amori L Guidetti P Muchowski PJ Schwarcz R. Dysfunctional kynurenine pathway metabolism in the R6/2 mouse model of Huntington’s disease. J Neurochem (2010) 113:1416–25. 10.1111/j.1471-4159.2010.06675.x 20236387 PMC3721540

[92] Campesan S Green EW Breda C Sathyasaikumar KV Muchowski PJ Schwarcz R The kynurenine pathway modulates neurodegeneration in a *Drosophila* model of Huntington’s disease. Curr Biol (2011) 21:961–6. 10.1016/j.cub.2011.04.028 21636279 PMC3929356

[93] Mazarei G Budac DP Lu G Adomat H Tomlinson Guns ES Moller T Age-dependent alterations of the kynurenine pathway in the YAC128 mouse model of Huntington disease. J Neurochem (2013) 127:852–67. 10.1111/jnc.12350 23786539

[94] Moroni F Lombardi G Robitaille Y Etienne P. Senile dementia and Alzheimer’s disease: lack of changes of the cortical content of quinolinic acid. Neurobiol Aging (1986) 7:249–53. 10.1016/0197-4580(86)90003-5 2944022

[95] Mourdian MM Heyes MP Pan JB Heuser IJ Markey SP Chase TN No changes in central quinolinic acid levels in Alzheimer’s disease. Neurosci Lett (1989) 105:233–8. 10.1016/0304-3940(89)90043-8 2535006

[96] Tohgi H Abe T Takahashi S Kimura M Takahashi J Kikuchi T. Concentrations of serotonin and its related substances in the cerebrospinal fluid in patients with Alzheimer type dementia. Neurosci Lett (1992) 141:9–12. 10.1016/0304-3940(92)90322-X 1508406

[97] Baran H Jellinger K Deecke L. Kynurenine metabolism in Alzheimer’s disease. J Neural Transm (1999) 106:165–81. 10.1007/s007020050149 10226937

[98] Hartai Z Juhasz A Rimanoczy A Janaky T Donko T Dux L Decreased serum and red blood cell kynurenic acid levels in Alzheimer’s disease. Neurochem Int (2007) 50:308–13. 10.1016/j.neuint.2006.08.012 17023091

[99] Gulaj E Pawlak K Bien B Pawlak D. Kynurenine and its metabolites in Alzheimer’s disease patients. Adv Med Sci (2010) 55:204–11. 10.2478/v10039-010-0023-6 20639188

[100] Zwilling D Huang SY Sathyasaikumar KV Notarangelo FM Guidetti P Wu HQ Kynurenine 3-monooxygenase inhibition in blood ameliorates neurodegeneration. Cell (2011) 145:863–74. 10.1016/j.cell.2011.05.020 21640374 PMC3118409

[101] Schwarz MJ Guillemin GJ Teipel SJ Buerger K Hampel H. Increased 3-hydroxykynurenine serum concentrations differentiate Alzheimer’s disease patients from controls. Eur Arch Psychiatry Clin Neurosci (2013) 263:345–52. 10.1007/s00406-012-0384-x 23192697

[102] Frazer A Pandey GN Mendels J Metabolism of tryptophan in depressive disease. Arch Gen Psychiatry (1973) 29:528–35. 10.1001/archpsyc.1973.04200040070012 4748313

[103] Myint AM Kim YK Verkerk R Scharpe S Steinbusch H Leonard B. Kynurenine pathway in major depression: evidence of impaired neuroprotection. J Affect Disord (2007) 98:143–51. 10.1016/j.jad.2006.07.013 16952400

[104] Erhardt S Lim CK Linderholm KR Janelidze S Lindqvist D Samuelsson M Connecting inflammation with glutamate agonism in suicidality. Neuropsychopharmacology (2013) 38:743–52. 10.1038/npp.2012.248 23299933 PMC3671988

[105] Bay-Richter C Linderholm KR Lim CK Samuelsson M Traskman-Bendz L Guillemin GJ A role for inflammatory metabolites as modulators of the glutamate N-methyl-d-aspartate receptor in depression and suicidality. Brain Behav Immun (2015) 43:110–7. 10.1016/j.bbi.2014.07.012 25124710

[106] Dahl J Andreassen OA Verkerk R Malt UF Sandvik L Brundin L Ongoing episode of major depressive disorder is not associated with elevated plasma levels of kynurenine pathway markers. Psychoneuroendocrinology (2015) 56:12–22. 10.1016/j.psyneuen.2015.02.011 25770856

[107] Savitz J Drevets WC Smith CM Victor TA Wurfel BE Bellgowan PS Putative neuroprotective and neurotoxic kynurenine pathway metabolites are associated with hippocampal and amygdalar volumes in subjects with major depressive disorder. Neuropsychopharmacology (2015) 40:463–71. 10.1038/npp.2014.194 25074636 PMC4443961

[108] Savitz J Drevets WC Wurfel BE Ford BN Bellgowan PS Victor TA Reduction of kynurenic acid to quinolinic acid ratio in both the depressed and remitted phases of major depressive disorder. Brain Behav Immun (2015) 46:55–9. 10.1016/j.bbi.2015.02.007 25686798 PMC4414807

[109] Wichers MC Koek GH Robaeys G Verkerk R Scharpe S Maes M. IDO and interferon-alpha-induced depressive symptoms: a shift in hypothesis from tryptophan depletion to neurotoxicity. Mol Psychiatry (2005) 10:538–44. 10.1038/sj.mp.4001600 15494706

[110] Van Gool AR Verkerk R Fekkes D Bannink M Sleijfer S Kruit WH Neurotoxic and neuroprotective metabolites of kynurenine in patients with renal cell carcinoma treated with interferon-alpha: course and relationship with psychiatric status. Psychiatry Clin Neurosci (2008) 62:597–602. 10.1111/j.1440-1819.2008.01854.x 18950381

[111] Miller CL Llenos IC Dulay JR Weis S Upregulation of the initiating step of the kynurenine pathway in postmortem anterior cingulate cortex from individuals with schizophrenia and bipolar disorder. Brain Res (2006) 107(3–1074):25–37. 10.1016/j.brainres.2005.12.056 16448631

[112] Myint AM Kim YK Verkerk R Park SH Scharpe S Steinbusch HW Tryptophan breakdown pathway in bipolar mania. J Affect Disord (2007) 102:65–72. 10.1016/j.jad.2006.12.008 17270276

[113] Olsson SK Samuelsson M Saetre P Lindstrom L Jonsson EG Nordin C Elevated levels of kynurenic acid in the cerebrospinal fluid of patients with bipolar disorder. J Psychiatry Neurosci (2010) 35:195–9. 10.1503/jpn.090180 20420770 PMC2861136

[114] Olsson SK Sellgren C Engberg G Landen M Erhardt S. Cerebrospinal fluid kynurenic acid is associated with manic and psychotic features in patients with bipolar I disorder. Bipolar Disord (2012) 14:719–26. 10.1111/bdi.12009 23030601

[115] Johansson AS Owe-Larsson B Asp L Kocki T Adler M Hetta J Activation of kynurenine pathway in ex vivo fibroblasts from patients with bipolar disorder or schizophrenia: cytokine challenge increases production of 3-hydroxykynurenine. J Psychiatr Res (2013) 47:1815–23. 10.1016/j.jpsychires.2013.08.008 24012176

[116] Savitz J Dantzer R Wurfel BE Victor TA Ford BN Bodurka J Neuroprotective kynurenine metabolite indices are abnormally reduced and positively associated with hippocampal and amygdalar volume in bipolar disorder. Psychoneuroendocrinology (2015) 52:200–11. 10.1016/j.psyneuen.2014.11.015 25486577 PMC4297593

[117] Schwarcz R Rassoulpour A Wu HQ Medoff D Tamminga CA Roberts RC. Increased cortical kynurenate content in schizophrenia. Biol Psychiatry (2001) 50:521–30. 10.1016/S0006-3223(01)01078-2 11600105

[118] Nilsson LK Linderholm KR Engberg G Paulson L Blennow K Lindstrom LH Elevated levels of kynurenic acid in the cerebrospinal fluid of male patients with schizophrenia. Schizophr Res (2005) 80:315–22. 10.1016/j.schres.2005.07.013 16125901

[119] Yao JK Dougherty GG Jr Reddy RD Keshavan MS Montrose DM Matson WR Altered interactions of tryptophan metabolites in first-episode neuroleptic-naive patients with schizophrenia. Mol Psychiatry (2010) 15:938–53. 10.1038/mp.2009.33 19401681 PMC2953575

[120] Condray R Dougherty GG Jr Keshavan MS Reddy RD Haas GL Montrose DM 3-Hydroxykynurenine and clinical symptoms in first-episode neuroleptic-naive patients with schizophrenia. Int J Neuropsychopharmacol (2011) 14:756–67. 10.1017/S1461145710001689 21275080 PMC3117924

[121] Myint AM Schwarz MJ Verkerk R Mueller HH Zach J Scharpe S Reversal of imbalance between kynurenic acid and 3-hydroxykynurenine by antipsychotics in medication-naive and medication-free schizophrenic patients. Brain Behav Immun (2011) 25:1576–81. 10.1016/j.bbi.2011.05.005 21620952

[122] Sathyasaikumar KV Stachowski EK Wonodi I Roberts RC Rassoulpour A Mcmahon RP Impaired kynurenine pathway metabolism in the prefrontal cortex of individuals with schizophrenia. Schizophr Bull (2011) 37:1147–56. 10.1093/schbul/sbq112 21036897 PMC3196941

[123] Linderholm KR Skogh E Olsson SK Dahl ML Holtze M Engberg G Increased levels of kynurenine and kynurenic acid in the CSF of patients with schizophrenia. Schizophr Bull (2012) 38:426–32. 10.1093/schbul/sbq086 20729465 PMC3329991

[124] Carlborg A Jokinen J Jonsson EG Erhardt S Nordstrom P. CSF kynurenic acid and suicide risk in schizophrenia spectrum psychosis. Psychiatry Res (2013) 205:165–7. 10.1016/j.psychres.2012.08.021 22980480

[125] Kegel ME Bhat M Skogh E Samuelsson M Lundberg K Dahl ML Imbalanced kynurenine pathway in schizophrenia. Int J Tryptophan Res (2014) 7:15–22. 10.4137/IJTR.S16800 25288889 PMC4179604

[126] Baran H Cairns N Lubec B Lubec G. Increased kynurenic acid levels and decreased brain kynurenine aminotransferase I in patients with down syndrome. Life Sci (1996) 58:1891–9. 10.1016/0024-3205(96)00173-7 8637415

[127] Mcfarlane HG Kusek GK Yang M Phoenix JL Bolivar VJ Crawley JN. Autism-like behavioral phenotypes in BTBR T+tf/J mice. Genes Brain Behav (2008) 7:152–63. 10.1111/j.1601-183X.2007.00330.x 17559418

[128] Oades RD Dauvermann MR Schimmelmann BG Schwarz MJ Myint AM. Attention-deficit hyperactivity disorder (ADHD) and glial integrity: S100B, cytokines and kynurenine metabolism – effects of medication. Behav Brain Funct (2010) 6:29. 10.1186/1744-9081-6-29 20509936 PMC2889842

[129] Coyle JT Schwarcz R Lesion of striatal neurones with kainic acid provides a model for Huntington’s chorea. Nature (1976) 263:244–6. 10.1038/263244a0 8731

[130] Stoy N Mackay GM Forrest CM Christofides J Egerton M Stone TW Tryptophan metabolism and oxidative stress in patients with Huntington’s disease. J Neurochem (2005) 93:611–23. 10.1111/j.1471-4159.2005.03070.x 15836620

[131] Browne SE Beal MF. Oxidative damage in Huntington’s disease pathogenesis. Antioxid Redox Signal (2006) 8:2061–73. 10.1089/ars.2006.8.2061 17034350

[132] Chang KH Wu YR Chen YC Chen CM. Plasma inflammatory biomarkers for Huntington’s disease patients and mouse model. Brain Behav Immun (2015) 44:121–7. 10.1016/j.bbi.2014.09.011 25266150

[133] Silvestroni A Faull RL Strand AD Moller T. Distinct neuroinflammatory profile in post-mortem human Huntington’s disease. Neuroreport (2009) 20:1098–103. 10.1097/WNR.0b013e32832e34ee 19590393

[134] Moller T. Neuroinflammation in Huntington’s disease. J Neural Transm (2010) 117:1001–8. 10.1007/s00702-010-0430-7 20535620

[135] Sapp E Kegel KB Aronin N Hashikawa T Uchiyama Y Tohyama K Early and progressive accumulation of reactive microglia in the Huntington disease brain. J Neuropathol Exp Neurol (2001) 60:161–72. 11273004 10.1093/jnen/60.2.161

[136] Pavese N Gerhard A Tai YF Ho AK Turkheimer F Barker RA Microglial activation correlates with severity in Huntington disease: a clinical and PET study. Neurology (2006) 66:1638–43. 10.1212/01.wnl.0000222734.56412.17 16769933

[137] Tai YF Pavese N Gerhard A Tabrizi SJ Barker RA Brooks DJ Microglial activation in presymptomatic Huntington’s disease gene carriers. Brain (2007) 130:1759–66. 10.1093/brain/awm044 17400599

[138] Franciosi S Ryu JK Shim Y Hill A Connolly C Hayden MR Age-dependent neurovascular abnormalities and altered microglial morphology in the YAC128 mouse model of Huntington disease. Neurobiol Dis (2012) 45:438–49. 10.1016/j.nbd.2011.09.003 21946335

[139] Goedert M Spillantini MG. A century of Alzheimer’s disease. Science (2006) 314:777–81. 10.1126/science.1132814 17082447

[140] Guillemin GJ Brew BJ Noonan CE Takikawa O Cullen KM. Indoleamine 2,3 dioxygenase and quinolinic acid immunoreactivity in Alzheimer’s disease hippocampus. Neuropathol Appl Neurobiol (2005) 31:395–404. 10.1111/j.1365-2990.2005.00655.x 16008823

[141] Xiang Z Haroutunian V Ho L Purohit D Pasinetti GM. Microglia activation in the brain as inflammatory biomarker of Alzheimer’s disease neuropathology and clinical dementia. Dis Markers (2006) 22:95–102. 10.1155/2006/276239 16410654 PMC3850819

[142] Guillemin GJ Williams KR Smith DG Smythe GA Croitoru-Lamoury J Brew BJ. Quinolinic acid in the pathogenesis of Alzheimer’s disease. Adv Exp Med Biol (2003) 527:167–76. 10.1007/978-1-4615-0135-0_19 15206729

[143] Rogawski MA Wenk GL. The neuropharmacological basis for the use of memantine in the treatment of Alzheimer’s disease. CNS Drug Rev (2003) 9:275–308. 10.1111/j.1527-3458.2003.tb00254.x 14530799 PMC6741669

[144] Farlow MR. NMDA receptor antagonists. A new therapeutic approach for Alzheimer’s disease. Geriatrics (2004) 59:22–7. 15224791

[145] Chang YT Chang WN Tsai NW Huang CC Kung CT Su YJ The roles of biomarkers of oxidative stress and antioxidant in Alzheimer’s disease: a systematic review. Biomed Res Int (2014) 2014:182303. 10.1155/2014/182303 24949424 PMC4053273

[146] Rahman A Ting K Cullen KM Braidy N Brew BJ Guillemin GJ. The excitotoxin quinolinic acid induces tau phosphorylation in human neurons. PLoS One (2009) 4:e6344. 10.1371/journal.pone.0006344 19623258 PMC2709912

[147] Serrano-Pozo A Mielke ML Gomez-Isla T Betensky RA Growdon JH Frosch MP Reactive glia not only associates with plaques but also parallels tangles in Alzheimer’s disease. Am J Pathol (2011) 179:1373–84. 10.1016/j.ajpath.2011.05.047 21777559 PMC3157187

[148] Edison P Archer HA Gerhard A Hinz R Pavese N Turkheimer FE Microglia, amyloid, and cognition in Alzheimer’s disease: an [11C](R)PK11195-PET and [11C]PIB-PET study. Neurobiol Dis (2008) 32:412–9. 10.1016/j.nbd.2008.08.001 18786637

[149] Oddo S Caccamo A Kitazawa M Tseng BP Laferla FM. Amyloid deposition precedes tangle formation in a triple transgenic model of Alzheimer’s disease. Neurobiol Aging (2003) 24:1063–70. 10.1016/j.neurobiolaging.2003.08.012 14643377

[150] Fang F Lue LF Yan S Xu H Luddy JS Chen D RAGE-dependent signaling in microglia contributes to neuroinflammation, Abeta accumulation, and impaired learning/memory in a mouse model of Alzheimer’s disease. FASEB J (2010) 24:1043–55. 10.1096/fj.09-139634 19906677 PMC3231946

[151] Rush AJ Trivedi MH Wisniewski SR Nierenberg AA Stewart JW Warden D Acute and longer-term outcomes in depressed outpatients requiring one or several treatment steps: a STAR*D report. Am J Psychiatry (2006) 163:1905–17. 10.1176/appi.ajp.163.11.1905 17074942

[152] Fukuda K. Etiological classification of depression based on the enzymes of tryptophan metabolism. BMC Psychiatry (2014) 14:372. 10.1186/s12888-014-0372-y 25540092 PMC4321701

[153] Eisenberger NI Berkman ET Inagaki TK Rameson LT Mashal NM Irwin MR. Inflammation-induced anhedonia: endotoxin reduces ventral striatum responses to reward. Biol Psychiatry (2010) 68:748–54. 10.1016/j.biopsych.2010.06.010 20719303 PMC3025604

[154] Grigoleit JS Kullmann JS Wolf OT Hammes F Wegner A Jablonowski S Dose-dependent effects of endotoxin on neurobehavioral functions in humans. PLoS One (2011) 6:e28330. 10.1371/journal.pone.0028330 22164271 PMC3229570

[155] Moussavi S Chatterji S Verdes E Tandon A Patel V Ustun B. Depression, chronic diseases, and decrements in health: results from the World Health Surveys. Lancet (2007) 370:851–8. 10.1016/S0140-6736(07)61415-9 17826170

[156] Raison CL Capuron L Miller AH. Cytokines sing the blues: inflammation and the pathogenesis of depression. Trends Immunol (2006) 27:24–31. 10.1016/j.it.2005.11.006 16316783 PMC3392963

[157] O’Connor JC Lawson MA Andre C Briley EM Szegedi SS Lestage J Induction of IDO by bacille Calmette-Guerin is responsible for development of murine depressive-like behavior. J Immunol (2009) 182:3202–12. 10.4049/jimmunol.0802722 19234218 PMC2664258

[158] Salazar A Gonzalez-Rivera BL Redus L Parrott JM O’Connor JC. Indoleamine 2,3-dioxygenase mediates anhedonia and anxiety-like behaviors caused by peripheral lipopolysaccharide immune challenge. Horm Behav (2012) 62:202–9. 10.1016/j.yhbeh.2012.03.010 22504306 PMC3425718

[159] Lawson MA Parrott JM Mccusker RH Dantzer R Kelley KW O’Connor JC. Intracerebroventricular administration of lipopolysaccharide induces indoleamine-2,3-dioxygenase-dependent depression-like behaviors. J Neuroinflammation (2013) 10:87. 10.1186/1742-2094-10-87 23866724 PMC3733827

[160] Heisler JM O’Connor JC. Indoleamine 2,3-dioxygenase-dependent neurotoxic kynurenine metabolism mediates inflammation-induced deficit in recognition memory. Brain Behav Immun (2015). 10.1016/j.bbi.2015.06.022 26130057 PMC4631688

[161] Rajkowska G Miguel-Hidalgo JJ. Gliogenesis and glial pathology in depression. CNS Neurol Disord Drug Targets (2007) 6:219–33. 10.2174/187152707780619326 17511618 PMC2918806

[162] Frick LR Williams K Pittenger C. Microglial dysregulation in psychiatric disease. Clin Dev Immunol (2013) 2013:608654. 10.1155/2013/608654 23690824 PMC3652125

[163] Steiner J Bielau H Brisch R Danos P Ullrich O Mawrin C Immunological aspects in the neurobiology of suicide: elevated microglial density in schizophrenia and depression is associated with suicide. J Psychiatr Res (2008) 42:151–7. 10.1016/j.jpsychires.2006.10.013 17174336

[164] Corona AW Norden DM Skendelas JP Huang Y O’Connor JC Lawson M Indoleamine 2,3-dioxygenase inhibition attenuates lipopolysaccharide induced persistent microglial activation and depressive-like complications in fractalkine receptor (CX(3)CR1)-deficient mice. Brain Behav Immun (2013) 31:134–42. 10.1016/j.bbi.2012.08.008 22926082 PMC3554840

[165] Henry CJ Huang Y Wynne A Hanke M Himler J Bailey MT Minocycline attenuates lipopolysaccharide (LPS)-induced neuroinflammation, sickness behavior, and anhedonia. J Neuroinflammation (2008) 5:15. 10.1186/1742-2094-5-15 18477398 PMC2412862

[166] Bowden CL. Strategies to reduce misdiagnosis of bipolar depression. Psychiatr Serv (2001) 52:51–5. 10.1176/appi.ps.52.1.51 11141528

[167] Martinez-Aran A Vieta E Reinares M Colom F Torrent C Sanchez-Moreno J Cognitive function across manic or hypomanic, depressed, and euthymic states in bipolar disorder. Am J Psychiatry (2004) 161:262–70. 10.1176/appi.ajp.161.2.262 14754775

[168] Atlas A Gisslen M Nordin C Lindstrom L Schwieler L. Acute psychotic symptoms in HIV-1 infected patients are associated with increased levels of kynurenic acid in cerebrospinal fluid. Brain Behav Immun (2007) 21:86–91. 10.1016/j.bbi.2006.02.005 16603336

[169] Berk M Dodd S Kauer-Sant’anna M Malhi GS Bourin M Kapczinski F Dopamine dysregulation syndrome: implications for a dopamine hypothesis of bipolar disorder. Acta Psychiatr Scand Suppl (2007) 116:41–9. 10.1111/j.1600-0447.2007.01058.x 17688462

[170] Cousins DA Butts K Young AH. The role of dopamine in bipolar disorder. Bipolar Disord (2009) 11:787–806. 10.1111/j.1399-5618.2009.00760.x 19922550

[171] Goldstein BI Kemp DE Soczynska JK Mcintyre RS. Inflammation and the phenomenology, pathophysiology, comorbidity, and treatment of bipolar disorder: a systematic review of the literature. J Clin Psychiatry (2009) 70:1078–90. 10.4088/JCP.08r04505 19497250

[172] Munkholm K Brauner JV Kessing LV Vinberg M. Cytokines in bipolar disorder vs. healthy control subjects: a systematic review and meta-analysis. J Psychiatr Res (2013) 47:1119–33. 10.1016/j.jpsychires.2013.05.018 23768870

[173] Soderlund J Olsson SK Samuelsson M Walther-Jallow L Johansson C Erhardt S Elevation of cerebrospinal fluid interleukin-1ss in bipolar disorder. J Psychiatry Neurosci (2011) 36:114–8. 10.1503/jpn.100080 21138659 PMC3044194

[174] Rao JS Harry GJ Rapoport SI Kim HW. Increased excitotoxicity and neuroinflammatory markers in postmortem frontal cortex from bipolar disorder patients. Mol Psychiatry (2010) 15:384–92. 10.1038/mp.2009.47 19488045 PMC2844920

[175] Stertz L Magalhaes PV Kapczinski F. Is bipolar disorder an inflammatory condition? The relevance of microglial activation. Curr Opin Psychiatry (2013) 26:19–26. 10.1097/YCO.0b013e32835aa4b4 23196997

[176] Lisman JE Coyle JT Green RW Javitt DC Benes FM Heckers S Circuit-based framework for understanding neurotransmitter and risk gene interactions in schizophrenia. Trends Neurosci (2008) 31:234–42. 10.1016/j.tins.2008.02.005 18395805 PMC2680493

[177] Aoyama N Takahashi N Saito S Maeno N Ishihara R Ji X Association study between kynurenine 3-monooxygenase gene and schizophrenia in the Japanese population. Genes Brain Behav (2006) 5:364–8. 10.1111/j.1601-183X.2006.00231.x 16716206

[178] Kozak R Campbell BM Strick CA Horner W Hoffmann WE Kiss T Reduction of brain kynurenic acid improves cognitive function. J Neurosci (2014) 34:10592–602. 10.1523/JNEUROSCI.1107-14.2014 25100593 PMC6802596

[179] Webster MJ O’Grady J Kleinman JE Weickert CS. Glial fibrillary acidic protein mRNA levels in the cingulate cortex of individuals with depression, bipolar disorder and schizophrenia. Neuroscience (2005) 133:453–61. 10.1016/j.neuroscience.2005.02.037 15885920

[180] Fillman SG Cloonan N Catts VS Miller LC Wong J Mccrossin T Increased inflammatory markers identified in the dorsolateral prefrontal cortex of individuals with schizophrenia. Mol Psychiatry (2013) 18:206–14. 10.1038/mp.2012.110 22869038

[181] Rao JS Kim HW Harry GJ Rapoport SI Reese EA. Increased neuroinflammatory and arachidonic acid cascade markers, and reduced synaptic proteins, in the postmortem frontal cortex from schizophrenia patients. Schizophr Res (2013) 147:24–31. 10.1016/j.schres.2013.02.017 23566496 PMC3812915

[182] Hercher C Chopra V Beasley CL. Evidence for morphological alterations in prefrontal white matter glia in schizophrenia and bipolar disorder. J Psychiatry Neurosci (2014) 39:376–85. 10.1503/jpn.130277 24936776 PMC4214872

[183] Gos T Myint AM Schiltz K Meyer-Lotz G Dobrowolny H Busse S Reduced microglial immunoreactivity for endogenous NMDA receptor agonist quinolinic acid in the hippocampus of schizophrenia patients. Brain Behav Immun (2014) 41:59–64. 10.1016/j.bbi.2014.05.012 24886967

[184] Kenk M Selvanathan T Rao N Suridjan I Rusjan P Remington G Imaging neuroinflammation in gray and white matter in schizophrenia: an in-vivo PET study with [18F]-FEPPA. Schizophr Bull (2015) 41:85–93. 10.1093/schbul/sbu157 25385788 PMC4266311

[185] Najjar S Pearlman DM. Neuroinflammation and white matter pathology in schizophrenia: systematic review. Schizophr Res (2015) 161:102–12. 10.1016/j.schres.2014.04.041 24948485

[186] Gogtay N Giedd JN Lusk L Hayashi KM Greenstein D Vaituzis AC Dynamic mapping of human cortical development during childhood through early adulthood. Proc Natl Acad Sci U S A (2004) 101:8174–9. 10.1073/pnas.0402680101 15148381 PMC419576

[187] Pocivavsek A Wu HQ Elmer GI Bruno JP Schwarcz R. Pre- and postnatal exposure to kynurenine causes cognitive deficits in adulthood. Eur J Neurosci (2012) 35:1605–12. 10.1111/j.1460-9568.2012.08064.x 22515201 PMC3773083

[188] Pocivavsek A Thomas MA Elmer GI Bruno JP Schwarcz R. Continuous kynurenine administration during the prenatal period, but not during adolescence, causes learning and memory deficits in adult rats. Psychopharmacology (Berl) (2014) 231:2799–809. 10.1007/s00213-014-3452-2 24590052 PMC4074218

[189] Alexander KS Pocivavsek A Wu HQ Pershing ML Schwarcz R Bruno JP. Early developmental elevations of brain kynurenic acid impair cognitive flexibility in adults: reversal with galantamine. Neuroscience (2013) 238:19–28. 10.1016/j.neuroscience.2013.01.063 23395862 PMC3622758

[190] Pershing ML Bortz DM Pocivavsek A Fredericks PJ Jorgensen CV Vunck SA Elevated levels of kynurenic acid during gestation produce neurochemical, morphological, and cognitive deficits in adulthood: implications for schizophrenia. Neuropharmacology (2015) 90:33–41. 10.1016/j.neuropharm.2014.10.017 25446576 PMC4731221

[191] Forrest CM Khalil OS Pisar M Darlington LG Stone TW. Prenatal inhibition of the tryptophan-kynurenine pathway alters synaptic plasticity and protein expression in the rat hippocampus. Brain Res (2013) 1504:1–15. 10.1016/j.brainres.2013.01.031 23353758

[192] Forrest CM Khalil OS Pisar M Mcnair K Kornisiuk E Snitcofsky M Changes in synaptic transmission and protein expression in the brains of adult offspring after prenatal inhibition of the kynurenine pathway. Neuroscience (2013) 254:241–59. 10.1016/j.neuroscience.2013.09.034 24076085

[193] Khalil OS Pisar M Forrest CM Vincenten MC Darlington LG Stone TW. Prenatal inhibition of the kynurenine pathway leads to structural changes in the hippocampus of adult rat offspring. Eur J Neurosci (2014) 39:1558–71. 10.1111/ejn.12535 24646396 PMC4368408

[194] Pisar M Forrest CM Khalil OS Mcnair K Vincenten MC Qasem S Modified neocortical and cerebellar protein expression and morphology in adult rats following prenatal inhibition of the kynurenine pathway. Brain Res (2014) 1576:1–17. 10.1016/j.brainres.2014.06.016 24956103

[195] Meyza KZ Defensor EB Jensen AL Corley MJ Pearson BL Pobbe RL The BTBR T+ tf/J mouse model for autism spectrum disorders-in search of biomarkers. Behav Brain Res (2013) 251:25–34. 10.1016/j.bbr.2012.07.021 22958973 PMC3529977

[196] Missault S Van Den Eynde K Vanden Berghe W Fransen E Weeren A Timmermans JP The risk for behavioural deficits is determined by the maternal immune response to prenatal immune challenge in a neurodevelopmental model. Brain Behav Immun (2014) 42:138–46. 10.1016/j.bbi.2014.06.013 24973728

[197] Van den Eynde K Missault S Fransen E Raeymaekers L Willems R Drinkenburg W Hypolocomotive behaviour associated with increased microglia in a prenatal immune activation model with relevance to schizophrenia. Behav Brain Res (2014) 258:179–86. 10.1016/j.bbr.2013.10.005 24129217

[198] Juckel G Manitz MP Brune M Friebe A Heneka MT Wolf RJ. Microglial activation in a neuroinflammational animal model of schizophrenia – a pilot study. Schizophr Res (2011) 131:96–100. 10.1016/j.schres.2011.06.018 21752601

[199] Ribeiro BM Do Carmo MR Freire RS Rocha NF Borella VC De Menezes AT Evidences for a progressive microglial activation and increase in iNOS expression in rats submitted to a neurodevelopmental model of schizophrenia: reversal by clozapine. Schizophr Res (2013) 151:12–9. 10.1016/j.schres.2013.10.040 24257517

[200] Liu XC Holtze M Powell SB Terrando N Larsson MK Persson A Behavioral disturbances in adult mice following neonatal virus infection or kynurenine treatment – role of brain kynurenic acid. Brain Behav Immun (2014) 36:80–9. 10.1016/j.bbi.2013.10.010 24140727 PMC3947209

[201] Zhu F Zhang L Ding YQ Zhao J Zheng Y. Neonatal intrahippocampal injection of lipopolysaccharide induces deficits in social behavior and prepulse inhibition and microglial activation in rats: implication for a new schizophrenia animal model. Brain Behav Immun (2014) 38:166–74. 10.1016/j.bbi.2014.01.017 24530999

[202] Suzuki K Sugihara G Ouchi Y Nakamura K Futatsubashi M Takebayashi K Microglial activation in young adults with autism spectrum disorder. JAMA Psychiatry (2013) 70:49–58. 10.1001/jamapsychiatry.2013.272 23404112

[203] Toledo-Sherman LM Prime ME Mrzljak L Beconi MG Beresford A Brookfield FA Development of a series of aryl pyrimidine kynurenine monooxygenase inhibitors as potential therapeutic agents for the treatment of Huntington’s disease. J Med Chem (2015) 58:1159–83. 10.1021/jm501350y 25590515

[204] Giorgini F Huang SY Sathyasaikumar KV Notarangelo FM Thomas MA Tararina M Targeted deletion of kynurenine 3-monooxygenase in mice: a new tool for studying kynurenine pathway metabolism in periphery and brain. J Biol Chem (2013) 288:36554–66. 10.1074/jbc.M113.503813 24189070 PMC3868768

